# Supplementary material for: Child maltreatment, early life socioeconomic disadvantage and all-cause mortality in mid-adulthood: findings from a prospective British birth cohort
Source: BMJ Open. 2021 Sep 22;11(9):e050914. doi: 10.1136/bmjopen-2021-050914 (PMC8461284; doi:10.1136/bmjopen-2021-050914)
Supplement: Supplementary data [file bmjopen-2021-050914supp001.pdf]

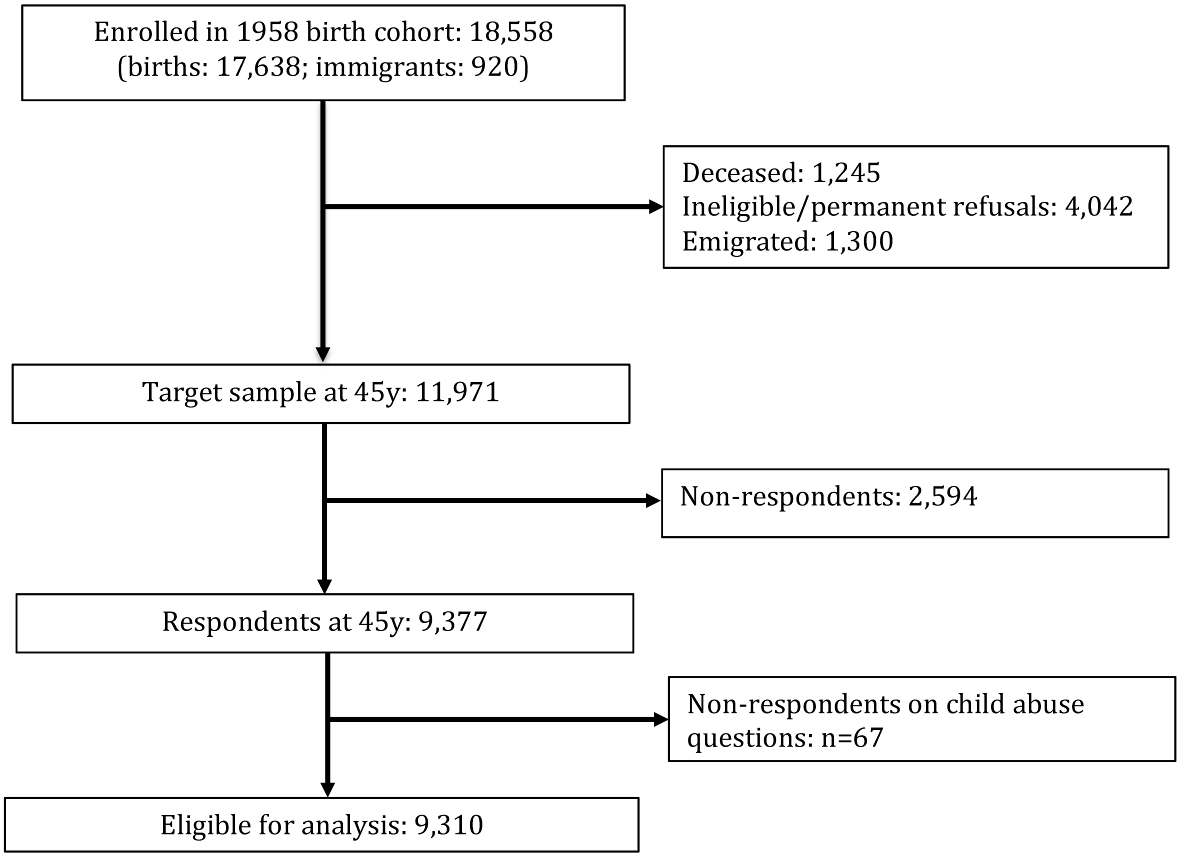

Supplementary Figure 1: Flow diagram of participants from birth who were eligible to be included in analytical sample
