# Supplementary material for: Child maltreatment, early life socioeconomic disadvantage and all-cause mortality in mid-adulthood: findings from a prospective British birth cohort
Source: BMJ Open. 2021 Sep 22;11(9):e050914. doi: 10.1136/bmjopen-2021-050914 (PMC8461284; doi:10.1136/bmjopen-2021-050914)
Supplement: Supplementary data [file bmjopen-2021-050914supp002.pdf]

Supplementary tables

Table S1. Distribution of potential intermediary variables (observed values)

Intermediary variables		N(%)/Mean(SD)
**Socioeconomic**		
33y social class[α]		
	I/II	3757 (42.2)
	III non-manual	1938 (21.8)
	III manual	1727 (19.4)
	IV/V	1476 (16.6)
33y educational qualifications		
	None	1765 (21.8)
	O-levels	2797 (34.5)
	A-levels	2424 (29.9)
	Degree	1123 (13.9)
**Behavioural**		
42y smoking		
	Never	4127 (45.8)
	Ex-smoker	2709 (30.1)
	Current	2180 (24.2)
45y problem drinking		
	Low risk	6262 (72.9)
	Risky/hazardous behaviour	1981 (23.1)
	High risk	213 (2.48)
	Almost certainly dependent	131 (1.53)
42y illegal drug use[£]		230 (2.56)
**Adiposity**		
45y obesity		2269 (24.5)
45y waist-hip ratio		0.87 (0.09)
**Mental health**		
42y psychological distress*[€]		2 (0,4)

**Cardio-metabolic factors**$^{\beta}$	
45y glycated haemoglobin (mmol/L)	5.25 (0.69)
45y triglycerides (mmol/L)*	1.6 (1.1,2.5)
45y low-density lipoprotein cholesterol (mmol/L)	3.45 (0.93)

N varies due to missing data. $^{\alpha}$classes I and II (professional/managerial), class III non-manual (skilled non-manual), class III manual (skilled manual) and classes IV and V (partly/unskilled manual); $^{£}$use of ecstasy, amphetamines, LSD, popper, magic mushrooms, cocaine, temazepan, crack, ketamine, heroin or methadone in last 12 months; *median(inter-quartile range); $^{€}$assessed using the malaise inventory (see Geoffroy et al. PLoS One 2013;8(11) for details); $^{\beta}$all adjusted for medications (see Li et al. BMJ Open 2019;9(3) for details)

Table S2. Potential intermediary variables and sex-adjusted risk of all-cause mortality (44/45y to 58y; N=9310)

Intermediary variable	HR (95%CI)
**Socioeconomic**	
33y social class [a]	1.21(1.09,1.34)
33y educational qualifications [a]	1.28(1.16,1.41)
**Behavioural**	
42y smoking [a]	3.01(2.32,3.89)
45y problem drinking [a]	3.04(1.65,5.62)
42y illegal drug use	2.59(1.62,4.14)
**Adiposity**	
45y obesity	1.38(1.08, 1.75)
45y waist-hip ratio [b]	2.32(0.44,12.2)
**Mental health**	
42y mental health[c]	1.11(1.07,1.15)
**Cardio-metabolic factors**	
45y glycated haemoglobin (mmol/L)	1.02(1.01,1.03)
45y triglycerides (mmol/L)	1.00(1.00,1.01)
45y low-density lipoprotein cholesterol (mmol/L)	1.00(1.00,1.01)

[a] For categorical variables, extreme category groups are compared (e.g.: lowest vs highest (reference group) social class);
[b] per 0.01 unit increase in waist/hip ratio; [c] per increase on 15-point malaise scale

Table S3. Hazard ratios (95% confidence intervals) for early-life adversities in relation to all-cause mortality in 1958 birth cohort participants aged 44/45y to 58y, men and women separately (N=9310)

	Model 1<br>HR (95%CI)	Model 2<br>HR (95%CI)	Model 3<br>HR (95%CI)
		Men	
Socioeconomic disadvantage	1.89 (1.26,2.84)	1.85 (1.22,2.78)	1.69 (1.12,2.57)
Neglect	1.81 (1.22,2.70)	1.49 (0.97,2.30)	1.45 (0.94,2.23)
Physical abuse	2.16 (1.36,3.45)	2.01 (1.25,3.23)	1.81 (1.00,3.29)
Psychological abuse	1.54 (0.98,2.40)	1.46 (0.93,2.28)	0.95 (0.54,1.68)
Witnessing abuse	1.82 (1.04,3.21)	1.66 (0.94,2.95)	1.13 (0.58,2.22)
Sexual abuse	5.41 (2.01,14.58)	5.51 (2.01,15.07)	4.37 (1.53,12.47)
		Women	
Socioeconomic disadvantage	2.60 (1.76,3.85)	2.44 (1.64,3.63)	2.23 (1.49,3.34)
Neglect	1.59 (0.98,2.57)	1.49 (0.89,2.48)	1.41 (0.84,2.35)
Physical abuse	2.57 (1.60,4.12)	2.33 (1.44,3.78)	1.67 (0.85,3.28)
Psychological abuse	1.73 (1.14,2.61)	1.66 (1.09,2.51)	1.02 (0.58,1.79)
Witnessing abuse	2.03 (1.27,3.26)	1.92 (1.19,3.10)	1.17 (0.64,2.14)
Sexual abuse	3.80 (2.15,6.73)	3.21 (1.79,5.75)	2.30 (1.18,4.49)

Adjustments as in Figure 1 footnotes.

Table S4. Correlation between early-life adversities (using Cramér's phi, observed data)

	Socioeconomic disadvantage	Neglect	Physical abuse	Psychological abuse	Witnessing abuse	Sexual abuse
Socioeconomic disadvantage		0.11	0.04	0.05	0.07	0.05
Neglect			0.07	0.04	0.07	0.04
Physical abuse				0.50	0.44	0.21
Psychological abuse					0.39	0.22
Witnessing abuse						0.25
Sexual abuse						

Table S5. Proportion of missing data (ascending order) in sample and distribution of observed and imputed analysis samples

Variable		Missing N(%)	Data distribution	
			Observed sample	Imputed sample*
			%/Mean	%/Mean
Sex		0 (0)		
	Males		49.6	49.6€
	Females		50.4	50.4€
Psychological abuse		0 (0)	10.7	10.7€
Physical abuse		2 (0.02)	6.04	6.04
Sexual abuse		2 (0.02)	1.60	1.60
Witnessing abuse		2 (0.02)	6.01	6.01
45y obesity		61 (0.66)	24.5	24.6
45y waist-to-hip ratio		64 (0.69)	0.87	0.87
Social class$^{\alpha}$ at birth		277 (2.98)		
	I/II		19.1	19.1
	III non-manual		10.0	10.0
	III manual		48.7	48.7
	IV/V/No male head		22.2	22.2
42y smoking		294 (3.16)		
	Never		45.8	45.7
	Ex		30.1	30.0
	Current		24.2	24.3
42y Malaise inventory		332 (3.57)	2.46	2.47
42y Illegal drug use		335 (3.60)	2.56	2.60
33y Adult social class$^{\alpha}$		412 (4.43)		
	I/II		42.2	41.7
	III non-manual		21.8	21.7
	III manual		19.4	19.5
	IV/V		16.6	17.1
Maternal age at birth (years)		496 (5.33)	27.5	27.5
45y Problem drinking		723 (7.77)		
	Low risk		72.9	73.2
	Risky/hazardous behaviour		23.1	22.8
	High risk		2.48	2.47

Almost certainly dependent		1.53	1.52
Child neglect	850 (9.13)	10.4	10.5
7y physical or cognitive impairment	1128 (12.1)	4.40	4.72
Birth order	1150 (12.4)		
1st born		38.5	38.8
2nd-4th		54.4	54.0
5th or more		7.17	7.18
7y tenure	1161 (12.5)		
Owner occupied		44.6	44.8
Council rented		37.6	37.5
Private rented		11.9	11.7
Other		5.87	5.99
7y Lacking household amenities	1190 (12.8)	17.0	16.9
33y educational attainment	1201 (12.9)		
None		10.0	10.8
Some		11.8	12.1
O-levels		34.5	34.4
A-levels		29.9	29.3
Degree		13.9	13.5
7y household crowding	1448 (15.6)	39.8	40.0
45y glycated haemoglobin (mmol/L)[£]	1478 (15.9)	165.1	165.2
45y Triglycerides (mmol/L)[£]	1545 (16.6)	52.6	52.9
Birthweight (adjusted for gestational age)[¥]	1581 (17.0)	0.03	0.02
45y low-density lipoprotein cholesterol (mmol/L)[£]	1950 (21.0)	120.1	120.9

*averaged over 20 imputed datasets; [€]No missing data on these variables; [α]classes I and II (professional/managerial), class III non-manual (skilled non-manual), class III manual (skilled manual) and classes IV and V (partly/unskilled manual); [£]glycated haemoglobin and all lipids are presented (and modelled in imputation models) as 100*ln(x) (to ensure data is normally distributed); [¥]standardised scale

Table S6. Hazard ratios (95% confidence intervals) for early-life adversities in relation to all-cause mortality in 1958 birth cohort participants aged 44/45y to 58y

	Model 1<br>HR (95%CI)	Model 2<br>HR (95%CI)	Model 3<br>HR (95%CI)
	Imputed data (N=9310)		
Socioeconomic disadvantage	2.22(1.68,2.94)	2.12(1.60,2.82)	1.93(1.45,2.58)
Neglect	1.71(1.26,2.33)	1.49(1.08,2.07)	1.43(1.03,1.98)
Physical abuse	2.35(1.69, 3.27)	2.15(1.54,3.02)	1.73(1.11,2.71)
Psychological abuse	1.64(1.21,2.22)	1.55(1.14,2.10)	0.99(0.66,1.47)
Witnessing abuse	1.94(1.35,2.79)	1.81(1.26,2.62)	1.15(0.73,1.80)
Sexual abuse	4.12(2.51,6.77)	3.60(2.18,5.96)	2.64(1.52,4.59)
	Complete case analysis[a]		
Socioeconomic disadvantage	2.22(1.68,2.94)	1.99(1.41,2.82)	1.78(1.25,2.54)
Neglect	1.72(1.27,2.34)	1.72(1.16,2.56)	1.68(1.13,2.50)
Physical abuse	2.35(1.69, 3.27)	2.40(1.59,3.64)	1.53(0.88,2.67)
Psychological abuse	1.64(1.21,2.22)	1.81(1.26,2.61)	1.08(0.67,1.75)
Witnessing abuse	1.94(1.35,2.79)	2.51(1.64,3.84)	1.56(0.92,2.62)
Sexual abuse	4.12(2.51,6.77)	5.64(3.10,10.26)	3.70(1.90,7.19)

[a]N varies from 8460 (neglect) to 9310 (psychological abuse) in Model 1 and 6645 (CMs) to 6922 (socioeconomic disadvantage) in Model 3 due to missing data. Adjustments as in Figure 1 footnotes.

Table S7. Hazard ratios (95% confidence intervals) for early-life adversities and risk of premature death (44/45y to 58y) adjusted separately for potential intermediaries[a] (N=9310).

	Socioeconomic disadvantage	Neglect	Physical abuse	Sexual abuse
Model 3[b]	1.96(1.47,2.61)	1.45 (1.04,2.03)	1.72(1.10, 2.70)	2.60(1.49, 4.52)
Socioeconomic				
+ 33y social class	1.89(1.42,2.53)	1.37(0.98,1.91)	1.74(1.11,2.73)	2.57(1.48,4.48)
+ 33y educational qualifications	1.82(1.36, 2.43)	1.29(0.92,1.80)	1.74(1.11,2.72)	2.56(1.48,4.45)
Behavioural				
+ 42y smoking,	1.75(1.31,2.34)	1.29(0.93,1.80)	1.57(1.00,2.45)	2.33(1.34,4.05)
+45y problem drinking	1.91(1.42,2.55)	1.45(1.04,2.01)	1.69(1.08,2.63)	2.78(1.60,4.83)
+42y illegal drug use	1.92(1.44, 2.57)	1.43(1.03,1.98)	1.68(1.07,2.62)	2.61(1.50,4.55)
Adiposity				
+ 45y obesity	1.92(1.44,2.56)	1.42(1.02, 1.97)	1.72(1.10,2.69)	2.63(1.51,4.58)
+ 45y waist-hip ratio	1.89(1.42,2.53)	1.39(1.00,1.92)	1.75(1.12,2.73)	2.73(1.57,4.76)
Mental Health				
+42y psychological distress	1.91(1.43,2.55)	1.37(0.98,1.91)	1.70(1.09,2.64)	2.54(1.46, 4.42)
Cardio-metabolic factors				
+ 45y glycated haemoglobin	1.89(1.41,2.52)	1.39 (1.00,1.93)	1.65(1.05, 2.58)	2.72(1.57, 4.73)
+ 45y triglycerides	1.92(1.44,2.57)	1.42(1.02,1.96)	1.72(1.10, 2.70)	2.67(1.53, 4.64)
+ 45y low-density lipoprotein cholesterol	1.93(1.45,2.58)	1.43 (1.03,1.98)	1.72(1.10, 2.69)	2.61(1.50, 4.54)

[a] For each pathway, models were adjusted for factors separately (not simultaneously). See text and Table S1 for details on intermediary factors.
[b] Model 3 (adjustments shown in Figure 1 footnotes)

Table S8: Hazard ratios (95% confidence intervals) for childhood socioeconomic disadvantage and neglect in relation to all-cause mortality in 1958 birth cohort participants aged 11y to 58y (N=15,092).

	Model 1 HR (95%CI)	Model 2 HR (95%CI)
Socioeconomic disadvantage	1.65(1.40,1.94)	1.55(1.31,1.83)
Neglect	1.72(1.46,2.01)	1.46(1.23,1.73)

Adjustments as in Figure 1 footnotes.

Table S9. Hazard ratios (95% confidence intervals) for distinct non-overlapping early-life adversities (versus none) in relation to all-cause mortality in 1958 birth cohort participants aged 44/45y to 58y.

Early Life adversities vs no Early life adversities	N[a](%)	Model 1 HR (95%CI)	Model 2 HR (95%CI)
No Early life adversities	5968 (78.5)	ref	ref
Socioeconomic disadvantage only	544 (7.16)	2.34(1.63,3.37)	2.30(1.59, 3.32)
Neglect[b] only	522 (7.26)	2.11(1.44,3.08)	2.01(1.34, 3.02)
Physical abuse only	66 (0.87)	1.73(0.60, 5.02)	1.66(0.57, 4.83)
Psychological abuse only	342 (4.50)	1,16(0.63,2.13)	1.17(0.63,2.15)
Witnessing abuse only	108 (1.42)	0.66(0.16,2.66)	0.65(0.16, 2.61)
Sexual abuse only	23 (0.30)	3.62(0.90,14.6)	3.70 (0.91, 15.0)

[a] N varies due to missing data; [b] Those with complete data on 6 or more of 11 neglect items; Adjustments as in Figure 1 footnotes.
