# Supplementary material for: Child maltreatment, early life socioeconomic disadvantage and all-cause mortality in mid-adulthood: findings from a prospective British birth cohort
Source: BMJ Open. 2021 Sep 22;11(9):e050914. doi: 10.1136/bmjopen-2021-050914 (PMC8461284; doi:10.1136/bmjopen-2021-050914)
Supplement: Author's manuscript [file bmjopen-2021-050914.draft_revisions.pdf]

BMJ Open is committed to open peer review. As part of this commitment we make the peer review history of every article we publish publicly available.

When an article is published we post the peer reviewers' comments and the authors' responses online. We also post the versions of the paper that were used during peer review. These are the versions that the peer review comments apply to.

The versions of the paper that follow are the versions that were submitted during the peer review process. They are not the versions of record or the final published versions. They should not be cited or distributed as the published version of this manuscript.

BMJ Open is an open access journal and the full, final, typeset and author-corrected version of record of the manuscript is available on our site with no access controls, subscription charges or pay-per-view fees (http://bmjopen.bmj.com).

If you have any questions on BMJ Open's open peer review process please email info.bmjopen@bmj.com

Child maltreatment, early life socioeconomic disadvantage and all-cause mortality in mid-adulthood: findings from a prospective British birth cohort

Journal:	*BMJ Open*
Manuscript ID	bmjopen-2021-050914
Article Type:	Original research
Date Submitted by the Author:	04-Mar-2021
Complete List of Authors:	Rogers, Nina; University College London, UCL Division of Surgery & Interventional Science<br>Power, Christine<br>Pinto Pereira, Snehal; UCL, UCL Division of Surgery & Interventional Science
Keywords:	PUBLIC HEALTH, Community child health < PAEDIATRICS, PREVENTIVE MEDICINE

SCHOLARONE™
Manuscripts

I, the Submitting Author has the right to grant and does grant on behalf of all authors of the Work (as defined in the below author licence), an exclusive licence and/or a non-exclusive licence for contributions from authors who are: i) UK Crown employees; ii) where BMJ has agreed a CC-BY licence shall apply, and/or iii) in accordance with the terms applicable for US Federal Government officers or employees acting as part of their official duties; on a worldwide, perpetual, irrevocable, royalty-free basis to BMJ Publishing Group Ltd ("BMJ") its licensees and where the relevant Journal is co-owned by BMJ to the co-owners of the Journal, to publish the Work in this journal and any other BMJ products and to exploit all rights, as set out in our licence.

The Submitting Author accepts and understands that any supply made under these terms is made by BMJ to the Submitting Author unless you are acting as an employee on behalf of your employer or a postgraduate student of an affiliated institution which is paying any applicable article publishing charge ("APC") for Open Access articles. Where the Submitting Author wishes to make the Work available on an Open Access basis (and intends to pay the relevant APC), the terms of reuse of such Open Access shall be governed by a Creative Commons licence – details of these licences and which Creative Commons licence will apply to this Work are set out in our licence referred to above.

Other than as permitted in any relevant BMJ Author's Self Archiving Policies, I confirm this Work has not been accepted for publication elsewhere, is not being considered for publication elsewhere and does not duplicate material already published. I confirm all authors consent to publication of this Work and authorise the granting of this licence.

For peer review only

Child maltreatment, early life socioeconomic disadvantage and all-cause mortality in mid-adulthood: findings from a prospective British birth cohort

Nina Trivedy Rogers (PhD)[1], Chris Power (PhD)[2], and Snehal M Pinto Pereira (PhD)[1*]

*Correspondence to: snehal.pereira@ucl.ac.uk, *Telephone: +44(0)20 7679 8336*

[1] UCL Division of Surgery & Interventional Science

[2] UCL Great Ormond Street Institute of Child Health, London WC1N 1EH, UK

Manuscript word count: 3286

For peer review only - http://bmjopen.bmj.com/site/about/guidelines.xhtml

Abstract

Objectives: Early-life adversities such as child maltreatment (neglect and abuse) and socioeconomic disadvantage have been associated with adult mortality. However, evidence is sparse for specific types of early-life adversity. We aimed to establish whether specific early-life adversities (i.e. different types of child maltreatment and socioeconomic disadvantage) were associated independently with all-cause mortality in mid-adulthood and to examine potential intermediary pathways.

Design: Prospective cohort study

Setting: 1958 British birth cohort: a longitudinal, population-based sample of individuals born in Great Britain during a single week in March 1958.

Participants: 9310 males and females with data on child maltreatment and mortality (44/45y to 58y).

Outcome measures: Mortality follow-up from 2002/3 to 2016 when participants were aged 44/45y to 58y. Death was ascertained via the NHS Central Register (N=296) or cohort maintenance activities (N=16).

Results: Prevalence of early-life adversities ranged from 1.6% (sexual abuse) to 11% (psychological abuse). Several, but not all, early-life adversities were associated with increased risk of premature death, independent of covariates and other adversities; adjusted hazard ratios were 2.64(95%CI:1.52,4.59) for sexual abuse, 1.93(1.45,2.58) for socioeconomic disadvantage, 1.73(1.11,2.71) for physical abuse and 1.43(1.03,1.98) for neglect. After adjustment for covariates and other adversities, no associations with mortality were observed for psychological and witnessing abuse. Regarding potential intermediaries (including child-to-adult height growth, adult socioeconomic factors, behaviours, adiposity, mental health and cardio-metabolic

For peer review only - http://bmjopen.bmj.com/site/about/guidelines.xhtml

markers), most associations attenuated after accounting for adult health behaviours (particularly smoking). In addition, early-life socioeconomic disadvantage and neglect associations attenuated after accounting for adult socioeconomic factors. The association for sexual abuse and premature mortality was largely unaffected by potential intermediaries.

Conclusions: Associations with premature mortality varied by type of early-life adversity: associations for sexual and physical abuse, neglect and socioeconomic disadvantage were independent of each other.

Keywords: Birth cohort, Child maltreatment, Child abuse, Child neglect, Early-life socioeconomic disadvantage, Life course epidemiology, premature mortality

Funding: UK Medical Research Council, US National Institute on Aging, UK Economic and Social Research Council and the Biotechnology and Biological Sciences Research Council

For peer review only

Strengths and limitations of this study

- Data were from a large population-based cohort followed from birth, which allowed prospective ascertainment of child neglect, early-life socioeconomic disadvantage, important covariates and potential intermediary factors.
- Information on different types of child abuse was reported retrospectively at 45y.
- Data on cause-specific mortality was not available; however, mortality data was collected over 14 years.

For peer review only

For peer review only - http://bmjopen.bmj.com/site/about/guidelines.xhtml

Background

Early-life adversities (ELAs) such as child maltreatment (neglect and abuse) and socioeconomic disadvantage are major public health issues[1,2]. These adversities are not uncommon, for example in the UK, approximately 9% of children and 22% of adolescents are estimated to experience neglect and/or physical, psychological or sexual abuse[3], and approximately 4.6 million children live in poverty[4]. Although a growing literature links child maltreatment (CM) to several poor health outcomes[5–7] in childhood through to older ages, evidence on links to mortality in adulthood is based primarily on adversity scores[8–11], which include other experiences such as living in a household with someone who has previously been imprisoned. While a more extensive literature including systemic reviews[2] demonstrates associations for early-life socioeconomic disadvantage, few studies[9] consider both CM and early-life socioeconomic disadvantage simultaneously in relation to mortality in adulthood. This omission is important because, although these ELAs are related, they represent distinct concepts[12] with potential differences in mortality risk. Clarifying the extent to which CM associates with later mortality independent of childhood socioeconomic background (and vice versa) would inform understanding of the role of different types of early-life exposures.

Moreover, it is possible that associations vary for *specific* types of CM and mortality in adulthood, given the differences reported for several outcomes in adulthood, including socioeconomic disadvantage[13,14], mental[15–17] and physical[6] health. Variation in associations with adult mortality cannot be determined from the sparse literature available to date for specific types of CM. One US study that combined multiple CMs together, found no risk of premature mortality although follow-up was limited to young adulthood[18]. In a second US study, associations were found in women but not men for physical and psychological abuse with all-cause mortality over 20-years of follow-up from ages 25y to 74y at baseline; information on sexual abuse and neglect was unavailable[9]. Thus, previous studies have investigated CM as a combined score of different types[9,18] or a limited number of types examined separately[9]. With such limitations of research conducted to date, possible differential associations for specific types of CM (i.e. their independence from each other as well as from early-life socioeconomic disadvantage) on mortality in adulthood are not well understood.

With respect to potential pathways from ELAs to adult mortality, it is well-established that, for example, CM is associated with detrimental factors in adulthood, including socioeconomic circumstances[13,14], risky health behaviors (e.g. smoking, drug misuse, problem drinking)[16,19,20], obesity[6], poor mental health[15–17] and poor physical health and development (e.g. child-to-adult height growth[21]). In turn,

For peer review only - http://bmjopen.bmj.com/site/about/guidelines.xhtml

For peer review only

these factors are linked to mortality[22–24]. Yet existing literature examining such intermediaries is limited, particularly in relation to CM. Understanding pathways through which specific types of ELAs link to mortality in mid-adulthood is important for developing appropriate interventions that aim to reduce inequalities in mortality.

Given current knowledge gaps, we aimed to establish in a general population sample followed from birth: (i) the extent to which CM and early-life socioeconomic disadvantage are associated with premature mortality in midlife (from 44/45y to 58y) and whether associations vary by type of ELA; and (ii) whether associations are explained by potential intermediaries including adult socioeconomic, behavioural, adiposity, mental health, cardio-metabolic status and child-to-adult height growth.

Methods

The 1958 British birth cohort consists of over 17,000 participants followed-up since birth during one week of March 1958[25]. Respondents in mid-adulthood are broadly representative of the surviving cohort[26]. Ethical approval was given, including at 50y by the London Multicenter Research Ethics Committee and participants gave informed consent at various sweeps. Of 11,971 invited at 44/45y, 9,310 completed questions on CM and had information on mortality (44/45-58y) (see supplementary figure 1).

Early-life adversities: Socioeconomic disadvantage was identified from prospectively recorded information on father's occupation at the participant's birth. Those with a father in an unskilled manual occupation were classified as disadvantaged. Child neglect was identified from prospectively collected information at 7y and 11y from interview questions to the child's mother and teacher. Eleven indicators of neglect were selected to represent conventional definitions and were summed to create a score (range 0-11). A score ≥3 yielded a prevalence estimate in line with other UK estimates[1,3], and was used here to define child neglect. Childhood (0-16y) physical, psychological, witnessing and sexual abuse was reported retrospectively at 44/45y using a confidential computer-assisted data-entry questionnaire. Child neglect and abuse measures have been used in several previous studies that, reassuringly, provide extensive evidence of construct validity[27]. Details of all ELAs are given in Table 1.

All-cause mortality: Information on deaths between 2002/3 and end of 2016 was ascertained from a variety of sources, mostly (N=296) through receipt of death certificates (including date of death) from the National Health Service Central Register. Information from relatives or close friends during survey

For peer review only - http://bmjopen.bmj.com/site/about/guidelines.xhtml

activities/cohort maintenance allowed identification of 16 further deaths (details in Table 2 footnotes). Cause-specific data were not available.

Covariates: were selected a-priori. All were prospectively recorded, including maternal age at birth, birthweight (adjusted for gestational age), birth order and 7y physical or cognitive impairment. Additional covariates for CM analyses included social class at birth and household factors (amenities, tenure and crowding) at 7y (details in Table 3 footnotes). Birthweight was ascertained from clinical records, parents reported all other factors.

Potential mid-adult intermediary factors: were selected based on established associations with both ELAs and premature mortality. Details of included factors are given in Table 4 footnotes, i.e. for adult (i) socioeconomic factors: social class and educational qualifications, (ii) behavioural factors: smoking, problem drinking and illegal drugs use in the last 12 months; (iii) adiposity: obesity and waist-hip ratio; (iv) mental health; (v) cardio-metabolic factors: glycated haemoglobin (HbA1c), triglycerides and low-density lipoprotein cholesterol (LDL-c), all adjusted for medications, and (vi) child-to-adult height trajectories. Cardio-metabolic factors, height, weight, hip and waist were measured by trained professionals; other factors were self-reported. Most considered intermediaries were associated with mortality in this cohort (Table S1).

Statistical analysis

Cox proportional hazard models were used to estimate hazard ratios and 95% confidence intervals (HR(95%CI)) for associations between each type of ELA and mortality. Survival time included the time from completion of the 44/45y questionnaire to the date of death, censoring (last date of contact) or the end of the study period (December 2016), whichever came first. Schoenfeld residuals were examined to test the assumption of proportional hazards for covariates and potential intermediaries; none violated the assumption (p-values≥0.12).

We examined associations between each type of ELA and mortality in separate analyses for men and women and also tested interactions with sex in analyses of both sexes combined. There was little evidence of effect modification, hence in a first level of analyses we adjusted for sex (model 1). Second, to assess whether associations were independent of other early-life factors, we additionally adjusted for covariates listed above (model 2). Third, because different types of ELAs often cluster[28], we assessed two-way

For peer review only - http://bmjopen.bmj.com/site/about/guidelines.xhtml

correlations between examined ELAs. Most ELAs were weakly or only modestly correlated (phi coefficient <0.50). Therefore, in model 3, we adjusted for all types of ELA simultaneously. To assess the role of potential intermediaries (socioeconomic, behavioural, adiposity, mental health, cardio-metabolic factors and child-to-adult height trajectories) in explaining ELA—mortality associations, we further adjusted model 3 for each potential intermediary separately.

In sensitivity analyses, we checked whether restricting the sample to those completing the CM questions at 44/45y affected results, by repeating analyses using the larger sample available for child neglect and socioeconomic disadvantage (N=15,092). Survival time included the time from completion of the 11y survey to the date of death, censoring or the end of the study period, whichever came first. Main findings were largely unaltered (Table S2) and we present findings for ELAs using the sample with complete data on CM at 44/45y (N= 9,310). As an additional check on the independence of associations for different types of ELAs from model 3, we examined associations with mortality for groups with only one specific type of ELA vs no ELA. Findings were broadly similar (albeit with wider confidence intervals) to main results from model 3 (Table S3).

Missing data ranged from 0.3% (social class at birth) to 20.9% (LDL-c). Data loss was minimized, by imputing missing data using multiple imputation chained equations. Imputation models included all model variables and main predictors of missingness[26]. Regression analyses were run across 20 imputed datasets and overall estimates obtained. Imputed results were similar to those obtained using observed values; the former are presented.

Patient and public Involvement

Patients and the public were not involved in the design of the study, or in the interpretation or writing up of the manuscript.

Results

The prevalence of ELAs varied from 1.6% (sexual abuse) to 11% (psychological abuse) with 10% classified as socioeconomically disadvantaged in early-life (Table 2). The majority of participants reported no ELA (71%) with 19% reporting one and 10% reporting two or more types of ELA. Between 44/45y and 58y, 3.4% of the sample died (N=312).

All types of ELA were associated with risk of death (44/45y-58y) after controlling for covariates (Model 2; Table 3), for example HR for neglect was 1.49(1.08,2.07) and for physical abuse was 2.15(1.54,3.02). In

For peer review only - http://bmjopen.bmj.com/site/about/guidelines.xhtml

models simultaneously adjusted for all other types of ELA (Model 3; Table 3) associations remained for all except psychological and witnessing abuse, namely for neglect (HR:1.43(1.03,1.98)), physical abuse (HR:1.73(1.11,2.71)), sexual abuse (HR:2.64(1.52,4.59)) and socioeconomic disadvantage (HR:1.93(1.45,2.58)). The reduction in HRs between Models 2 and 3 was seen consistently for all ELAs, although modest in some instances, for example, for early-life socioeconomic disadvantage the HR reduced from 2.12(1.60,2.82) to 1.93(1.45,2.58) after adjusting for all CMs.

In regard to potential intermediaries, associations between ELAs and death in mid-adulthood were largely unaffected by adjustment for the range of factors examined (Table 4). However, most associations attenuated after adjustment for adult health behaviors, for example HRs for physical abuse attenuated from 1.73(1.11,2.71) to 1.50(0.96,2.34). Separate adjustment for each health behavior in turn showed a predominant attenuating effect of smoking (Table S4). Additionally, associations for neglect and early-life socioeconomic disadvantage attenuated after controlling for adult socioeconomic factors. For sexual abuse and early-life socioeconomic disadvantage reductions in the strong associations with mortality in mid-adulthood were negligible after accounting for intermediaries.

Discussion

In this large population-based study on different types of ELA and mortality in mid-adulthood we showed several important findings. First, some ELAs, but not all, were associated with higher risk of premature mortality in mid-adulthood. That is, findings varied by type of adversity. Child sexual abuse was strongly associated with mortality with a 2.6 times higher risk of premature death in mid-adulthood, although it was the least prevalent adversity. Whilst for early-life socioeconomic disadvantage, an approximate doubling in risk of premature mortality applied to one in 10 of the population. For physical abuse and neglect the estimated elevated risk of death was more modest (73% and 43% higher respectively), whereas no associations were observed for psychological and witnessing abuse. Second, observed associations were independent of potential confounding factors and the other adversities examined. Importantly, the specific CM associations were mostly robust when accounting for early-life socioeconomic disadvantage and vice versa. Third in relation to potential intermediaries, associations for all types of ELA attenuated after controlling for adult health behaviours, in particular smoking. But, in some instances this attenuation was minor, such that for sexual abuse the association was largely unaltered. Associations for early-life socioeconomic disadvantage and neglect were also attenuated by adult socioeconomic factors. Whereas, other examined intermediaries including cardio-metabolic markers did little to explain observed

For peer review only - http://bmjopen.bmj.com/site/about/guidelines.xhtml

associations between specific CMs and mortality in mid-adulthood or for early-life socioeconomic disadvantage.

Our study has several notable strengths. The range of data available on different types of ELA facilitated simultaneous analysis to inform on their independent effects. This is essential for investigating distinct effects of CM on mortality, i.e. separate from those of socioeconomic background and also, in regard to specific types of CMs. Inclusion of child neglect is particularly important given that it is often ignored in research on CM[29]. A follow-up of approximately 14y is a further study strength, as is use of linked mortality data, which is independent of ongoing study participation. Alongside the 14y mortality follow-up there are advantages of using a single-age sample in reducing the range of possible causes of premature death and related underlying pathways. However, study limitations are acknowledged. Ascertainment of childhood maltreatment is not straightforward, with limitations noted for all methods[1], including those used here. While, child neglect indicators were measured prospectively and included many aspects of the conventional definition (e.g. failure to ensure a child's basic physical, emotional and educational needs), there were some omissions (e.g. failure to ensure a child's safety). However, our measure uses information from different sources (parents and teachers) which may reduce misclassification and rather than relying on individual items, we used a composite score. Abuse by a parent was reported retrospectively and does not include abuse by others possibly leading to an underestimate of prevalence. Nonetheless, prevalence estimates of CM were generally in keeping with previous approximates for the UK[1,3]. An exception is child sexual abuse where prevalence is low and estimates may be under-powered. Thus, we have used both prospective (neglect and early-life socioeconomic disadvantage) and retrospective (abuse) measures and we acknowledge that these may identify different groups of individuals[30]. However, it is reassuring that a broad range of studies based on our measures of child neglect and abuse provide extensive evidence of construct validity[27]. As with any long-term study, selection bias needs to be considered: by 45y, when information was collected on child abuse, not all in the cohort had survived (6.7% had died); however over half of these deaths had occurred before 7y (mostly in the first months of life)[26]. Selection bias may affect findings reported here, but only if patterns of association with mortality differ in the surviving and deceased populations. Relatedly, sensitivity analysis for child neglect and socioeconomic disadvantage in a larger sample with longer follow-up from 11y suggests that study results are robust. Finally, cause-specific mortality data was unavailable, restricting understanding of possible mechanisms linking ELAs to different causes of premature death.

Our main finding of varying associations for specific types of ELAs with risk of premature mortality is novel largely because there is a dearth of literature that focuses on such variations. The large population and

For peer review only - http://bmjopen.bmj.com/site/about/guidelines.xhtml

range of ELA measures examined in our study compared with two previous studies[9,18] has facilitated this novel finding. Notably, in respect of CMs, we found that sexual and physical abuse and also neglect were associated with elevated mortality in mid-adulthood in the 1958 birth cohort, but there were no associations for psychological and witnessing abuse. The strong association for child sexual abuse, 2.6 times higher risk of premature mortality, is particularly important given the lack of evidence to date. One previous study of CM and mortality did not include sexual abuse[9] and a second study considered sexual abuse, physical abuse and neglect as a composite measure[18]; thus comparison with our findings for specific types of CMs is not possible. The latter composite measure study reported no association with mortality in young adulthood[18] whereas, our focus is on premature mortality for the age range 44/45y to 58y. The life-stage examined might explain discrepant findings i.e. associations with mortality were not present in young adulthood[18] but may emerge by mid-adulthood as suggested here. Whereas for physical abuse, our finding of a 73% higher risk of premature mortality is consistent with a previous estimate of 58% higher risk of death for severe physical abuse in US women aged over 45y to 94y at the end of follow-up[9]. This broad similarity in estimates for physical abuse was unexpected given the wider age range of US study participants compared with our range 44/45y to 58y for mortality follow-up. Nonetheless, discrepant with our findings was the lack of a physical abuse association with mortality in US men[9], possibly due to known variations in main causes of death by age and gender[31]. For child neglect we are unable to compare our finding of an independent association, with a 43% higher risk of premature death in mid-adulthood, as neither of the two previous CM—mortality studies investigated this exposure separately[9,18]. Thus, our finding provides new evidence for an important component of CM where knowledge of long-term outcome is particularly sparse[29]. For witnessing abuse in childhood we are not aware of any previous study with which to confirm our null finding in relation to premature mortality; whereas for psychological abuse, findings for the US (weak association in women only[9]) and our UK (null) study are discrepant. Possible reasons for discrepancies include differences in age at death, abuse measurement and also, the extent to which other ELAs were taken into account. In respect of the latter, it is noteworthy that our findings for specific CM associations with elevated mortality in mid-adulthood were independent of other types of CM as well as childhood socioeconomic circumstances, highlighting the potential for long-term harm associated with specific CMs.

A further novelty of our study is the demonstration that the early-life socioeconomic disadvantage association of an approximate doubling in risk of premature all-cause mortality was independent of specific CMs. While links between early-life socioeconomic disadvantage and mortality in adulthood are well-established[2] and consistent with previous work in this cohort[32], few studies[9] consider both CM and early-life socioeconomic disadvantage simultaneously. By suggesting that, notwithstanding the utility of

For peer review only - http://bmjopen.bmj.com/site/about/guidelines.xhtml

understanding the long-term impact of CM, the latter does not appear to undermine or explain the strong and robust findings relating to childhood socioeconomic disadvantage our study adds new knowledge to the literature. This is important in a policy context as the recent emphasis on adverse childhood experiences may displace attention away from the early socioeconomic environment, as argued elsewhere[12].

Our findings suggest that adult smoking is a consistent and in some instances important explanatory factor across observed associations. This was expected because smoking remains one of the most common preventable causes of premature death in adults[33]; and, CM[6,20] and early-life socioeconomic disadvantage[34] are associated with subsequent smoking patterns. Thus, interventions to reduce smoking prevalence in specific ELA groups, either by reducing initiation or promoting cessation, might be considered as possible strategies to lessen differences in premature mortality. Interestingly, while specific CMs in this cohort were associated with the wide range of potential intermediary factors examined, these did not appear to explain associations with mortality. In particular, the strong association for sexual abuse was little explained by examined factors. Nonetheless, the potential intermediary factors considered here may play a role in pathways to mortality at older ages. Whereas in relation to the focus here on premature mortality, further insight into pathways from sexual abuse and other ELAs might be gained in future studies of cause-specific mortality.

In summary, our findings of independent associations for specific types of CM (sexual and physical abuse and neglect) and early-life socioeconomic disadvantage with increased risk of premature mortality in mid-adulthood highlight the long-lasting consequences of these ELAs. Smoking may be a particularly important intermediary for physical abuse, neglect and early-life socioeconomic disadvantage associations; adult socioeconomic factors may be an additional intermediary for neglect and early-life socioeconomic disadvantage. These findings are relevant for public health because, for example, an estimated 3.1 million adults in England and Wales reported being victims of sexual abuse before 16y[35] and approximately 4.6 million children in the UK live in poverty[4]. Moreover, relative child poverty is projected to rise from 29.7% to 36.6% in the UK between 2018 to 2022[36]. Given these stark projections and our study findings of a strong relationship between childhood disadvantage and an early adult death, policies focused on improving socioeconomic opportunities and assistance to adopt and maintain positive health behaviors for individuals from disadvantaged backgrounds may reduce the burden of premature mortality.

Conclusions

For peer review only - http://bmjopen.bmj.com/site/about/guidelines.xhtml

In sum, our findings highlight the potential of specific types of CMs (i.e. sexual abuse, physical abuse and neglect) for long-term harm. Notwithstanding this important finding, childhood socioeconomic disadvantage associations with premature mortality are strong and not explained by associations with CM.

Abbreviations

CM: Child maltreatment

ELA: Early-life adversity

For peer review only

For peer review only - http://bmjopen.bmj.com/site/about/guidelines.xhtml

Contributors

CP and SMPP conceived the study. NTR carried out the analysis and drafted the paper. All authors contributed to the interpretation of data, revision of the manuscript, and approved its final version.

Declaration of interests

We declare no competing interests.

Acknowledgments

Application for all-cause mortality was made through the UK Data service. This work was supported by US National Institute on Aging (NIA) of the National Institutes of Health under award number U24AG047867 and the UK Economic and Social Research Council (ESRC) and the Biotechnology and Biological Sciences Research Council (BBSRC) and by the National Institute for Health Research Biomedical Research Centre at Great Ormond Street Hospital for Children NHS Foundation Trust and University College London. SMPP is funded by a UK Medical Research Council Career Development Award (ref: MR/P020372/1). The views expressed in the publication are those of the authors and not necessarily those of the funders. The funders had no input into study design; data collection, analysis, and interpretation; in the writing of the report; and in the decision to submit the article for publication. Researchers were independent of influence from study funders.

Ethical declarations

Ethical approval was given, including at 50y by the London Multicenter Research Ethics Committee. Participants gave informed consent at various sweeps.

Data sharing statement Cohort data comply with ESRC data sharing policies, readers can access these data via the UK Data Archive at http://www.data-archive.ac.uk/

For peer review only

Table 1. Definition of early-life adversities (child maltreatment and early-life socioeconomic disadvantage) and representative variables from the 1958 British Birth cohort

	Definition[a]	1958 cohort variables	Age of ascertainment (method)[b]
Prospective report, birth to 11y			
Socioeconomic disadvantage (birth)		Based on father's occupation at birth[c], using the Registrar General's Classification. Fathers with an unskilled manual occupation or households with no male head were classified as disadvantaged.	Birth (P)
Neglect[d] (7y & 11y)	Failure to meet a child's basic physical, emotional, medical/dental, or education need; failure to provide adequate nutrition, hygiene, or shelter; or failure to ensure a child's safety	- Child looks undernourished, scruffy or dirty<br>- Mother never, or hardly ever takes child out[e]<br>- Father never, or hardly ever takes child out[e]<br>- Mother shows little or no interest in child's educational progress<br>- Father shows little or no interest in child's educational progress<br>-Mother and Father never, or hardly ever read to, or reads with the child	7 & 11y (T)<br>7 & 11y (P)<br>7 & 11y (P)<br>7 & 11y (T)<br>7 & 11y (T)<br>7y (P)
Retrospective report at 44/45y			
Physical abuse (0-16y)	Intentional use of physical force or implements against a child that results in, or has the potential to result in, physical injury.	I was physically abused by a parent – punched, kicked or hit or beaten with an object, or needed medical treatment	45y (S)
Psychological abuse[f] (0-16y)	Intentional behaviour that conveys to a child that s/he is worthless, flawed, unloved, unwanted, endangered, or valued only in meeting another's needs.<br>*UK definition[g] includes harmful (unintentional) parent-child interactions: 'the persistent emotional maltreatment of a child such as to cause severe and persistent adverse effects on the child's emotional development'*	- I was verbally abused by a parent (or parent-figure)<br>- I suffered humiliation, ridicule, bullying or mental cruelty from a parent (or parent-figure)<br>- Mother (or mother-figure) and father (or father-figure) were not at all affectionate	45y (S)
Witnessing abuse (0-16y)	Any incident of threatening behaviour, violence, or abuse (psychological, physical, sexual, financial, or emotional) between intimate partners or adult family members, irrespective of sex or sexuality	I witnessed physical or sexual abuse of others in my family	45y (S)
Sexual abuse (0-16y)	Any completed or attempted sexual act, sexual contact, or non-contact sexual interaction with a child by a caregiver	I was sexually abused by a parent (or parent-figure)	45y (S)

For peer review only - http://bmjopen.bmj.com/site/about/guidelines.xhtml

a: Gilbert et al. Lancet. 2009;373; b: (S): self-report; (T): teacher-report; (P): parent-report; c: socioeconomic position was classified as missing for fathers' who were unemployed or sick; d:Questions relating to child neglect at 7y and 11y were answered by the child's teacher and mother (or father if the mother was unavailable). The 11 neglect indicators were summed to create a score (range 0–11); those scoring > 3 were classified as neglected (see text for further details); e: e.g. walks, outings, picnics, visits, shopping; [f]In the 1958 cohort psychological abuse was defined as experiencing at least one of the three listed variables; g: Department for Education. Working together to safeguard children. Her Majesty's Government, 2006.

For peer review only

Table 2. Prevalence of early-life adversities and mortality[a] in the 1958 British birth cohort (N=9310).

Early-life adversity	Population sample N[b]	Total cases N (%)	Males (%)	Females (%)	Deaths N (%)
Socioeconomic disadvantage	9033	925 (10.2)	9.63	10.8	61 (6.59)
Neglect[c]	8460	878 (10.4)	11.1	9.69	49 (5.58)
Physical abuse	9308	562 (6.04)	5.93	6.14	40 (7.12)
Psychological abuse	9310	1000 (10.7)	8.87	12.6	50 (5.00)
Witnessing abuse	9308	559 (6.01)	4.42	7.57	33 (5.90)
Sexual abuse	9308	149 (1.60)	0.48	2.71	17 (11.4)
Deaths 44/45y-58y[a]	9310	312 (3.35)	3.68	3.03	

[a] Date of death was ascertained through receipt of death certificates to the Centre for Longitudinal Studies from the National Health Service Central Register (N=296) i.e. data missing for 16 individuals (see: National Child Development Study Deaths Dataset, 1958-2016 UK Data Service for details). Using survey/cohort maintenance data we determined if the deceased died between 45-50y (N=7), 50-55y (N=5) and 55-58y (N=4). Date of death was estimated as the mid-point between these ages.
[b] N varies due to missing data.
[c] Those with complete data on 6 or more of 11 neglect items.

For peer review only

.

Table 3: Hazard ratios (95% confidence intervals) for early-life adversities in relation to all-cause mortality in 1958 birth cohort participants aged 44/45y to 58y (N=9310).

	Model 1<br>HR (95%CI)	Model 2<br>HR (95%CI)	Model 3<br>HR (95%CI)
Socioeconomic disadvantage	2.22(1.68,2.94)	2.12(1.60,2.82)	1.93(1.45,2.58)
Neglect	1.71(1.26,2.33)	1.49(1.08,2.07)	1.43(1.03,1.98)
Physical abuse	2.35(1.69, 3.27)	2.15(1.54,3.02)	1.73(1.11,2.71)
Psychological abuse	1.64(1.21,2.22)	1.55(1.14,2.10)	0.99(0.66,1.47)
Witnessing abuse	1.94(1.35,2.79)	1.81(1.26,2.62)	1.15(0.73,1.80)
Sexual abuse	4.12(2.51,6.77)	3.60(2.18,5.96)	2.64(1.52,4.59)

Model 1: Adjusted for sex only.

Model 2: Additionally adjusted for maternal age at birth, birthweight (adjusted for gestational age), birth order and 7y physical or cognitive impairment (yes/no). For associations with neglect, physical abuse, psychological abuse, witnessing abuse and sexual abuse (but not for early-life disadvantage) models additionally adjusted for social class at birth (or if missing, at 7y), 7y household amenities (sharing or lack a bathroom, lavatory or hot water), 7y housing tenure (owner/occupier, renter or other), and 7y household crowding (1+ person/room).

Model 3: Model 2 plus simultaneous adjustments for all other early-life adversities.

For peer review only

Table 4. Early-life adversities and risk of all-cause mortality (Hazard ratio (95% CI)) in adults (44/45y to 58y) adjusted for mid-adult (i) socioeconomic (ii) behavioural, (iii) adiposity, (iv) mental health, (v) cardio-metabolic factors and (vi) child-to-adult height trajectories[a].

	Socioeconomic disadvantage	Neglect	Physical abuse	Sexual abuse
Model 3	1.93(1.45,2.58)	1.43 (1.03,1.98)	1.73(1.11,2.71)	2.64(1.52,4.59)
+ mid-adult socioeconomic factors	1.82(1.36,2.43)	1.28(0.91,1.79)	1.74(1.11,2.73)	2.54(1.46,4.42)
+ mid-adult behavioural factors	1.75(1.31,2.34)	1.32(0.95,1.83)	1.50(0.96,2.34)	2.43(1.40,4.23)
+ mid-adult adiposity	1.90(1.42,2.53)	1.39(1.00,1.93)	1.75(1.12,2.73)	2.71(1.56,4.73)
+ mid-adult mental health	1.91(1.43,2.55)	1.37(0.99,1.91)	1.70(1.09,2.64)	2.54(1.46,4.42)
+ mid-adult cardio-metabolic factors	1.89(1.42,2.53)	1.39(1.00,1.93)	1.65(1.05,2.57)	2.71(1.56,4.72)
+ child-to-adult height trajectories	1.93(1.45,2.58)	1.42(1.03,1.98)	1.73(1.11,2.71)	2.63(1.51,4.58)

Model 3 (adjustments shown in Table 3 footnotes)

[a] Models were adjusted for each intermediary group of factors separately (not simultaneously). Socioeconomic factors include: 33y social class (professional/managerial, skilled non-manual, skilled manual and partly skilled/unskilled) and 33y educational qualifications (none, CSE/O-level, A-level, degree level or higher); behavioural factors include: 42y smoking (never, ex, light/moderate, heavy), 45y problem drinking (4 categories using AUDIT scale: low-risk, risky or hazardous behaviour, high risk and almost certainly dependent), 42y illegal drugs use (ecstasy, amphetamines, LSD, popper, magic mushrooms, cocaine, temazepan, crack, ketamine, heroin or methadone in last 12 months); adiposity included: 45y obesity (BMI ≥30 kg/m^2) and 45y waist-to-hip ratio; mental health factors included: 42y psychological distress assessed using the malaise inventory (see Geoffroy et al. PLoS One 2013;8(11) for details); cardio-metabolic factors included: 45y HbA1c, triglycerides and LDL-c, all adjusted for medications (see Li et al. BMJ Open 2019;9(3) for details); child-to-adult height trajectories included: 7y and 45y measured height (see Denholm R et al. Int J Epidemiol 2013;42(5) for details).

For peer review only

References

1. Gilbert R, Widom CS, Browne K, Fergusson D, Webb E, Janson S. Burden and consequences of child maltreatment in high-income countries. Lancet. 2009;373(9657):68–81.

2. Galobardes B, Lynch JW, Davey Smith G. Childhood Socioeconomic Circumstances and Cause-specific Mortality in Adulthood: Systematic Review and Interpretation. Epidemiol Rev. 2004;26(1):7–21.

3. Radford L, Corral S, Bradley C, Fisher H, Bassett C, Howat N, et al. Child abuse and neglect in the UK today. NSPCC, London. 2011.

4. Measuring Poverty 2019: A report of the Social Metrics Commission. The Legatum Institute. 2019.

5. Norman RE, Byambaa M, De R, Butchart A, Scott J, Vos T. The Long-Term Health Consequences of Child Physical Abuse, Emotional Abuse, and Neglect: A Systematic Review and Meta-Analysis. PLoS Med. 2012;9(11).

6. Power C, Pinto Pereira SM, Li L. Childhood Maltreatment and BMI Trajectories to Mid-Adult Life: Follow-Up to Age 50y in a British Birth Cohort. PLoS One. 2015;10(3).

7. Lanier P, Jonson-Reid M, Stahlschmidt MJ, Drake B, Constantino J. Child maltreatment and pediatric health outcomes: a longitudinal study of low-income children. J Pediatr Psychol. 2010;35(5):511–22.

8. Brown DW, Anda RF, Tiemeier H, Felitti VJ, Edwards VJ, Croft JB, et al. Adverse Childhood Experiences and the Risk of Premature Mortality. Am J Prev Med. 2009;37(5):389–96.

9. Chen E, Turiano NA, Mroczek DK, Miller GE. Association of Reports of Childhood Abuse and All-Cause Mortality Rates in Women. JAMA Psychiatry. 2016;73(9):920–7.

10. Bellis MA, Hughes K, Leckenby N, Hardcastle KA, Perkins C, Lowey H. Measuring mortality and the burden of adult disease associated with adverse childhood experiences in England: a national survey. J Public Health (Bangkok). 2015;37(3):445–54.

11. Felitti VJ, Anda RF, Nordenberg D, Williamson DF, Spitz AM, Edwards V, et al. Relationship of childhood abuse and household dysfunction to many of the leading causes of death in adults. The Adverse Childhood Experiences (ACE) Study. Am J Prev Med. 1998;14(4):245–58.

12. Taylor-Robinson DC, Straatmann VS, Whitehead M. Adverse childhood experiences or adverse childhood socioeconomic conditions? Lancet Public Heal. 2018;3(6).

13. Pinto Pereira SM, Li L, Power C. Child Maltreatment and Adult Living Standards at 50 Years. Pediatrics. 2017;139(1).

14. Currie J, Spatz Widom C. Long-Term Consequences of Child Abuse and Neglect on Adult Economic Well-Being. Child Maltreat. 2010;15(2):111–20.

15. Kisely S, Abajobir AA, Mills R, Strathearn L, Clavarino A, Najman JM. Child maltreatment and mental health problems in adulthood: birth cohort study. Br J Psychiatry. 2018;213(6):698–703.

For peer review only

16. Rehan W, Antfolk J, Johansson A, Jern P, Santtila P. Experiences of severe childhood maltreatment, depression, anxiety and alcohol abuse among adults in Finland. PLoS One. 2017;12(5).

17. Geoffroy M-C, Pereira SP, Li L, Power C. Child Neglect and Maltreatment and Childhood-to-Adulthood Cognition and Mental Health in a Prospective Birth Cohort. J Am Acad Child Adolesc Psychiatry. 2016;55(1):33–40.

18. White HR, Widom CS. Does childhood victimization increase the risk of early death? A 25-year prospective study. Child Abuse Negl. 2003;27(7):841–53.

19. Spatz Widom C, Marmorstein NR, Raskin White H. Childhood victimization and illicit drug use in middle adulthood. Psychol Addict Behav. 2006;20(4):394–403.

20. Kisely S, Abajobir AA, Mills R, Strathearn L, Clavarino A, Gartner C, et al. Child Maltreatment and Persistent Smoking From Adolescence Into Adulthood: A Birth Cohort Study. Nicotine Tob Res. 2019;22(1):66–73.

21. Denholm R, Power C, Li L. Adverse childhood experiences and child-to-adult height trajectories in the 1958 British birth cohort. Int J Epidemiol. 2013;42(5):1399–409.

22. Walker ER, McGee RE, Druss BG. Mortality in Mental Disorders and Global Disease Burden Implications. JAMA Psychiatry. 2015;72(4):334.

23. Peacock A, Leung J, Larney S, Colledge S, Hickman M, Rehm J, et al. Global statistics on alcohol, tobacco and illicit drug use: 2017 status report. Addiction. 2018;113(10):1905–26.

24. Berrington de Gonzalez A, Hartge P, Cerhan JR, Flint AJ, Hannan L, MacInnis RJ, et al. Body-mass index and mortality among 1.46 million white adults. N Engl J Med. 2010;363(23):2211–9.

25. Power C, Elliott J. Cohort profile: 1958 British birth cohort (National Child Development Study). Int J Epidemiol. 2006;35(1):34–41.

26. Atherton K, Fuller E, Shepherd P, Strachan DP, Power C. Loss and representativeness in a biomedical survey at age 45 years: 1958 British birth cohort. J Epidemiol Community Health. 2008;62(3):216–23.

27. Power C, Li L, Pinto Pereira SM. Child maltreatment (neglect and abuse) in the 1958 birth cohort: an overview of associations with developmental trajectories and long-term outcomes. Longit Life Course Stud. 2020;

28. Dong M, Anda RF, Felitti VJ, Dube SR, Williamson DF, Thompson TJ, et al. The interrelatedness of multiple forms of childhood abuse, neglect, and household dysfunction. Child Abuse Negl. 2004;28(7):771–84.

29. Stoltenborgh M, Bakermans-Kranenburg MJ, van IJzendoorn MH. The neglect of child neglect: a meta-analytic review of the prevalence of neglect. Soc Psychiatry Psychiatr Epidemiol. 2013;48(3):345–55.

30. Baldwin JR, Reuben A, Newbury JB, Danese A. Agreement Between Prospective and Retrospective Measures of Childhood Maltreatment. JAMA Psychiatry. American Medical

For peer review only

Association; 2019 Jun 1;76(6):584.

31. Mortality team. Does our sex affect what we die from? Off Natl Stat. 2016;

32. Power C, Hyppönen E, Smith GD. Socioeconomic position in childhood and early adult life and risk of mortality: a prospective study of the mothers of the 1958 British birth cohort. Am J Public Health. 2005;95(8):1396–402.

33. Doll R, Peto R, Boreham J, Sutherland I. Mortality in relation to smoking: 50 years' observations on male British doctors. BMJ. 2004;328(7455):1519.

34. Tian J, Gall S, Patterson K, Otahal P, Blizzard L, Patton G, et al. Socioeconomic position over the life course from childhood and smoking status in mid-adulthood: results from a 25-year follow-up study. BMC Public Health. BioMed Central; 2019;19(1):169.

35. Child abuse in England and Wales: January 2020. Statistical Bulletin. Office for National Statistics. 2020.

36. Hood A, Waters T. Living standards, poverty and inequality in the UK: 2017–18 to 2021–22 [Internet]. 2017 [cited 2021 Jan 1]. p. Institute for Fiscal Studies. Available from: https://www.ifs.org.uk/publications/10028

For peer review only

Supplementary tables

Table S1. Potential intermediary variables and risk of all-cause mortality (44/45y to 58y) adjusted for sex

Intermediary variable	HR (95%CI)
Socioeconomic	
33y social class [a]	1.21(1.09,1.34)
33y educational qualifications [a]	1.28(1.16,1.41)
Behavioural	
42y smoking [a]	3.01(2.32,3.89)
45y problem drinking [a]	3.04(1.65,5.62)
42y illegal drug use	2.59(1.62,4.14)
Adiposity	
45y obesity [a]	1.38(1.08, 1.75)
45y waist-hip ratio [b]	2.32(0.44,12.2)
Mental health	
42y mental health [c]	1.11(1.07,1.15)
Cardio-metabolic factors	
45y glycated haemoglobin (mmol/L)	1.02(1.01,1.03)
45y triglycerides (mmol/L)	1.00(1.00,1.01)
45y low-density lipoprotein cholesterol (mmol/L)	1.00(1.00,1.01)
Child to adult height growth[d]	
7y height	0.89(0.76, 1.04)
7-45y height growth [d]	1.00(0.76, 1.32)

[a] For categorical variables, extreme category groups are compared (e.g.: lowest vs highest (reference group) social class)

[b] per 0.01 unit increase in waist/hip ratio

[c] per increase on 15-point malaise scale

[d] 7y height and growth (7y-45y) modelled simultaneously

For peer review only

Table S2: Hazard ratios (95% confidence intervals) for childhood socioeconomic disadvantage and neglect in relation to all-cause mortality in 1958 birth cohort participants aged 11y to58y (N=15,092).

	Model 1 HR (95%CI)	Model 2 HR (95%CI)
Socioeconomic disadvantage	1.65(1.40,1.94)	1.55(1.31,1.83)
Neglect	1.72(1.46,2.01)	1.46(1.23,1.73)

Model 1: Adjusted for sex only.

Model 2: Additionally adjusted for maternal age at birth, birthweight (adjusted for gestational age), birth order and 7y physical or cognitive impairment (yes/no). For associations with neglect models additionally adjusted for social class at birth (or if missing, at 7y), 7y household amenities (sharing or lack a bathroom, lavatory or hot water), 7y housing tenure (owner/occupier, renter or other), and 7y household crowding (1+ person/room).

For peer review only

Table S3 Hazard ratios (95% confidence intervals) for distinct non-overlapping early-life adversities (versus none) in relation to all-cause mortality in 1958 birth cohort participants aged 44/45y to 58y.

Early Life adversities vs no Early life adversities	N[a](%)	Model 1 HR (95%CI)	Model 2 HR (95%CI)
No Early life adversities	5968 (78.5)	ref	ref
Socioeconomic disadvantage only	544 (7.16)	2.34(1.63,3.37)	2.30(1.59, 3.32)
Neglect[b] only	522 (7.26)	2.11(1.44,3.08)	2.01(1.34, 3.02)
Physical abuse only	66 (0.87)	1.73(0.60, 5.02)	1.66(0.57, 4.83)
Psychological abuse only	342 (4.50)	1,16(0.63,2.13)	1.17(0.63,2.15)
Witnessing abuse only	108 (1.42)	0.66(0.16,2.66)	0.65(0.16, 2.61)
Sexual abuse only	23 (0.30)	3.62(0.90,14.6)	3.70 (0.91, 15.0)

[a] N varies due to missing data.
[b] Those with complete data on 6 or more of 11 neglect items.

Model 1: Adjusted for sex only.

Model 2: Additionally, adjusted for maternal age at birth, birthweight (adjusted for gestational age), birth order and 7y physical or cognitive impairment (yes/no). For associations with neglect, physical abuse, psychological abuse, witnessing abuse and sexual abuse (but not for early-life disadvantage) models additionally adjusted for social class at birth (or if missing, at 7y), 7y household amenities (sharing or lack a bathroom, lavatory or hot water), 7y housing tenure (owner/occupier, renter or other), and 7y household crowding (1+ person/room).

For peer review only

Table S4 Hazard ratios (95% confidence intervals) for early-life adversities and risk of premature death (44/45y to 58y) adjusted separately for potential intermediaries[a].

	Socioeconomic disadvantage	Neglect	Physical abuse	Sexual abuse
Model 3[b]	1.96(1.47,2.61)	1.45 (1.04,2.03)	1.72(1.10, 2.70)	2.60(1.49, 4.52)
Socioeconomic				
+ 33y social class	1.89(1.42,2.53)	1.37(0.98,1.91)	1.74(1.11,2.73)	2.57(1.48,4.48)
+ 33y educational qualifications	1.82(1.36, 2.43)	1.29(0.92,1.80)	1.74(1.11,2.72)	2.56(1.48,4.45)
Behavioural				
+ 42y smoking,	1.75(1.31,2.34)	1.29(0.93,1.80)	1.57(1.00,2.45)	2.33(1.34,4.05)
+45y problem drinking	1.91(1.42,2.55)	1.45(1.04,2.01)	1.69(1.08,2.63)	2.78(1.60,4.83)
+42y illegal drug use	1.92(1.44, 2.57)	1.43(1.03,1.98)	1.68(1.07,2.62)	2.61(1.50,4.55)
Adiposity				
+ 45y obesity	1.92(1.44,2.56)	1.42(1.02, 1.97)	1.72(1.10,2.69)	2.63(1.51,4.58)
+ 45y waist-hip ratio	1.89(1.42,2.53)	1.39(1.00,1.92)	1.75(1.12,2.73)	2.73(1.57,4.76)
Mental Health				
+42y psychological distress	1.91(1.43,2.55)	1.37(0.98,1.91)	1.70(1.09,2.64)	2.54(1.46, 4.42)
Cardio-metabolic factors				
+ 45y glycated haemoglobin	1.89(1.41,2.52)	1.39 (1.00,1.93)	1.65(1.05, 2.58)	2.72(1.57, 4.73)
+ 45y triglycerides	1.92(1.44,2.57)	1.42(1.02,1.96)	1.72(1.10, 2.70)	2.67(1.53, 4.64)
+ 45y low-density lipoprotein cholesterol	1.93(1.45,2.58)	1.43 (1.03,1.98)	1.72(1.10, 2.69)	2.61(1.50, 4.54)
Child and adult height				
+ 7y height	1.93(1.44,2.58)	1.43(1.03,1.98)	1.73(1.11,2.71)	2.63(1.51,4.58)
+ 45y height	1.93(1.45,2.58)	1.43(1.03,1.98)	1.73(1.11,2.71)	2.64(1.52,4.59)

[a] For each pathway, models were adjusted for factors separately (not simultaneously). See text and Table 4 for details on intermediary factors.

[b] Model 3 (adjustments shown in Table 3 footnotes)

For peer review only

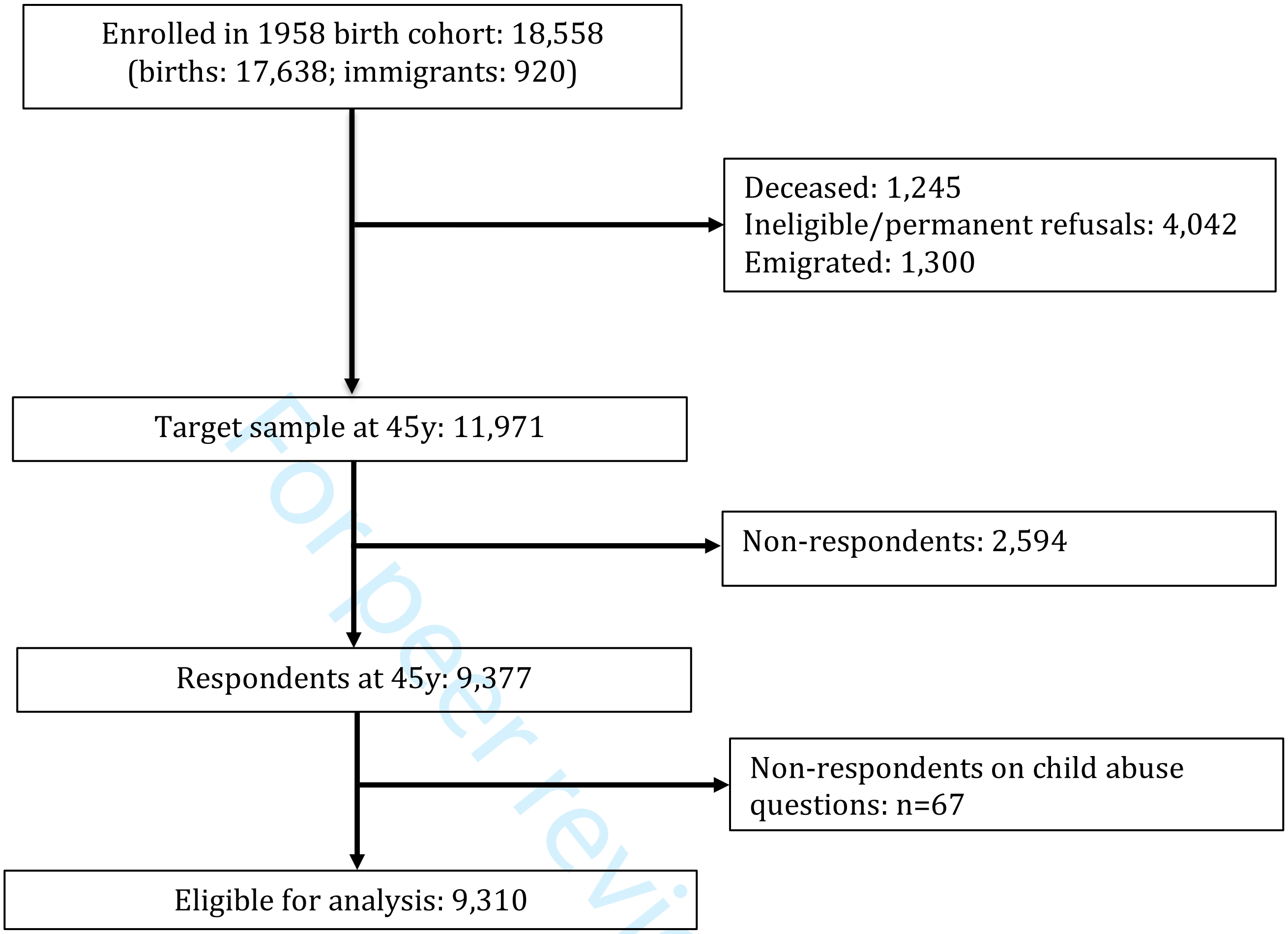

Supplementary Figure 1: Flow diagram of participants from birth who were eligible to be included in analytical sample

For peer review only

STROBE Statement—checklist of items that should be included in reports of observational studies

	Item No	Recommendation	Page No
**Title and abstract**	1	(*a*) Indicate the study's design with a commonly used term in the title or the abstract	1
		(*b*) Provide in the abstract an informative and balanced summary of what was done and what was found	2
**Introduction**			
Background/rationale	2	Explain the scientific background and rationale for the investigation being reported	5
Objectives	3	State specific objectives, including any prespecified hypotheses	6
**Methods**			
Study design	4	Present key elements of study design early in the paper	6
Setting	5	Describe the setting, locations, and relevant dates, including periods of recruitment, exposure, follow-up, and data collection	6
Participants	6	(*a*) *Cohort study*—Give the eligibility criteria, and the sources and methods of selection of participants. Describe methods of follow-up<br>*Case-control study*—Give the eligibility criteria, and the sources and methods of case ascertainment and control selection. Give the rationale for the choice of cases and controls<br>*Cross-sectional study*—Give the eligibility criteria, and the sources and methods of selection of participants	
		(*b*) *Cohort study*—For matched studies, give matching criteria and number of exposed and unexposed<br>*Case-control study*—For matched studies, give matching criteria and the number of controls per case	
Variables	7	Clearly define all outcomes, exposures, predictors, potential confounders, and effect modifiers. Give diagnostic criteria, if applicable	6-7
Data sources/ measurement	8*	For each variable of interest, give sources of data and details of methods of assessment (measurement). Describe comparability of assessment methods if there is more than one group	*6-8*
Bias	9	Describe any efforts to address potential sources of bias	8
Study size	10	Explain how the study size was arrived at	8
Quantitative variables	11	Explain how quantitative variables were handled in the analyses. If applicable, describe which groupings were chosen and why	8
Statistical methods	12	(*a*) Describe all statistical methods, including those used to control for confounding	8
		(*b*) Describe any methods used to examine subgroups and interactions	8
		(*c*) Explain how missing data were addressed	8
		(*d*) *Cohort study*—If applicable, explain how loss to follow-up was addressed<br>*Case-control study*—If applicable, explain how matching of cases and controls was addressed<br>*Cross-sectional study*—If applicable, describe analytical methods taking account of sampling strategy	
		(*e*) Describe any sensitivity analyses	8

Continued on next page

For peer review only - http://bmjopen.bmj.com/site/about/guidelines.xhtml

**Results**			
Participants	13*	(a) Report numbers of individuals at each stage of study—eg numbers potentially eligible, examined for eligibility, confirmed eligible, included in the study, completing follow-up, and analysed	9
		(b) Give reasons for non-participation at each stage	
		(c) Consider use of a flow diagram	
Descriptive data	14*	(a) Give characteristics of study participants (eg demographic, clinical, social) and information on exposures and potential confounders	9
		(b) Indicate number of participants with missing data for each variable of interest	
		(c) *Cohort study*—Summarise follow-up time (eg, average and total amount)	
Outcome data	15*	*Cohort study*—Report numbers of outcome events or summary measures over time	*9*
		Case-control study—Report numbers in each exposure category, or summary measures of exposure	
		Cross-sectional study—Report numbers of outcome events or summary measures	
Main results	16	(*a*) Give unadjusted estimates and, if applicable, confounder-adjusted estimates and their precision (eg, 95% confidence interval). Make clear which confounders were adjusted for and why they were included	9
		(*b*) Report category boundaries when continuous variables were categorized	
		(*c*) If relevant, consider translating estimates of relative risk into absolute risk for a meaningful time period	
Other analyses	17	Report other analyses done—eg analyses of subgroups and interactions, and sensitivity analyses	9
**Discussion**			
Key results	18	Summarise key results with reference to study objectives	9-10
Limitations	19	Discuss limitations of the study, taking into account sources of potential bias or imprecision. Discuss both direction and magnitude of any potential bias	10-11
Interpretation	20	Give a cautious overall interpretation of results considering objectives, limitations, multiplicity of analyses, results from similar studies, and other relevant evidence	11-12
Generalisability	21	Discuss the generalisability (external validity) of the study results	12
**Other information**			
Funding	22	Give the source of funding and the role of the funders for the present study and, if applicable, for the original study on which the present article is based	14

*Give information separately for cases and controls in case-control studies and, if applicable, for exposed and unexposed groups in cohort and cross-sectional studies.

Note: An Explanation and Elaboration article discusses each checklist item and gives methodological background and published examples of transparent reporting. The STROBE checklist is best used in conjunction with this article (freely available on the Web sites of PLoS Medicine at http://www.plosmedicine.org/, Annals of Internal Medicine at http://www.annals.org/, and Epidemiology at http://www.epidem.com/). Information on the STROBE Initiative is available at www.strobe-statement.org.

For peer review only

BMJ Open

Child maltreatment, early life socioeconomic disadvantage and all-cause mortality in mid-adulthood: findings from a prospective British birth cohort

Journal:	*BMJ Open*
Manuscript ID	bmjopen-2021-050914.R1
Article Type:	Original research
Date Submitted by the Author:	19-Jul-2021
Complete List of Authors:	Rogers, Nina; University College London, UCL Division of Surgery & Interventional Science<br>Power, Christine<br>Pinto Pereira, Snehal; UCL, UCL Division of Surgery & Interventional Science
**Primary Subject Heading**:	Public health
Secondary Subject Heading:	Paediatrics
Keywords:	PUBLIC HEALTH, Community child health < PAEDIATRICS, PREVENTIVE MEDICINE

SCHOLARONE™
Manuscripts

I, the Submitting Author has the right to grant and does grant on behalf of all authors of the Work (as defined in the below author licence), an exclusive licence and/or a non-exclusive licence for contributions from authors who are: i) UK Crown employees; ii) where BMJ has agreed a CC-BY licence shall apply, and/or iii) in accordance with the terms applicable for US Federal Government officers or employees acting as part of their official duties; on a worldwide, perpetual, irrevocable, royalty-free basis to BMJ Publishing Group Ltd ("BMJ") its licensees and where the relevant Journal is co-owned by BMJ to the co-owners of the Journal, to publish the Work in this journal and any other BMJ products and to exploit all rights, as set out in our licence.

The Submitting Author accepts and understands that any supply made under these terms is made by BMJ to the Submitting Author unless you are acting as an employee on behalf of your employer or a postgraduate student of an affiliated institution which is paying any applicable article publishing charge ("APC") for Open Access articles. Where the Submitting Author wishes to make the Work available on an Open Access basis (and intends to pay the relevant APC), the terms of reuse of such Open Access shall be governed by a Creative Commons licence – details of these licences and which Creative Commons licence will apply to this Work are set out in our licence referred to above.

Other than as permitted in any relevant BMJ Author's Self Archiving Policies, I confirm this Work has not been accepted for publication elsewhere, is not being considered for publication elsewhere and does not duplicate material already published. I confirm all authors consent to publication of this Work and authorise the granting of this licence.

For peer review only

Child maltreatment, early life socioeconomic disadvantage and all-cause mortality in mid-adulthood: findings from a prospective British birth cohort

Nina Trivedy Rogers (PhD)[1], Chris Power (PhD)[2], and Snehal M Pinto Pereira (PhD)[1*]

*Correspondence to: snehal.pereira@ucl.ac.uk, *Telephone: +44(0)20 7679 8336*

[1] UCL Division of Surgery & Interventional Science

[2] UCL Great Ormond Street Institute of Child Health, London WC1N 1EH, UK

Manuscript word count: 3519

For peer review only

For peer review only - http://bmjopen.bmj.com/site/about/guidelines.xhtml

Abstract

Objectives: Early-life adversities (ELAs) such as child maltreatment (neglect and abuse) and socioeconomic disadvantage have been associated with adult mortality. However, evidence is sparse for specific types of ELA. We aimed to establish whether specific ELAs (i.e. different types of child maltreatment and socioeconomic disadvantage) were associated independently with all-cause mortality in mid-adulthood and to examine potential intermediary pathways.

Design: Prospective cohort study

Setting: 1958 British birth cohort: a longitudinal, population-based sample of individuals born in Great Britain during a single week in March 1958.

Participants: 9310 males and females with data on child maltreatment and mortality (44/45y to 58y).

Outcome measures: Mortality follow-up from 2002/3 to 2016 when participants were aged 44/45y to 58y. Death was ascertained via the NHS Central Register (N=296) or cohort maintenance activities (N=16).

Results: Prevalence of ELAs ranged from 1.6% (sexual abuse) to 11% (psychological abuse). Several, but not all, ELAs were associated with increased risk of premature death, independent of covariates and other adversities; adjusted hazard ratios were 2.64(95%CI:1.52,4.59) for sexual abuse, 1.93(1.45,2.58) for socioeconomic disadvantage, 1.73(1.11,2.71) for physical abuse and 1.43(1.03,1.98) for neglect. After adjustment for covariates and other adversities, no associations with mortality were observed for psychological and witnessing abuse. Regarding potential intermediaries (including adult socioeconomic factors, behaviours, adiposity, mental health and cardio-metabolic markers), most associations attenuated after accounting for adult health behaviours (particularly smoking). In addition, early-life socioeconomic disadvantage and

For peer review only - http://bmjopen.bmj.com/site/about/guidelines.xhtml

neglect associations attenuated after accounting for adult socioeconomic factors. The association for sexual abuse and premature mortality was largely unaffected by potential intermediaries.

Conclusions: Associations with premature mortality varied by type of ELA: associations for sexual and physical abuse, neglect and socioeconomic disadvantage were independent of each other. Different types of ELAs could influence premature mortality via different pathways; this requires further research.

Keywords: Birth cohort, Child maltreatment, Child abuse, Child neglect, Early-life socioeconomic disadvantage, Life course epidemiology, premature mortality

Funding: UK Medical Research Council, US National Institute on Aging, UK Economic and Social Research Council and the Biotechnology and Biological Sciences Research Council

For peer review only

For peer review only - http://bmjopen.bmj.com/site/about/guidelines.xhtml

Strengths and limitations of this study

- Data were from a large population-based cohort followed from birth, which allowed prospective ascertainment of child neglect, early-life socioeconomic disadvantage, important covariates and potential intermediary factors.
- Information on different types of child abuse was reported retrospectively at 45y.
- Data on cause-specific mortality was not available; however, mortality data was collected over 14 years.

For peer review only

For peer review only - http://bmjopen.bmj.com/site/about/guidelines.xhtml

Background

Early-life adversities (ELAs) such as child maltreatment (neglect and abuse) and socioeconomic disadvantage are major public health issues[1,2]. These adversities are not uncommon, for example in the UK, approximately 9% of children and 22% of adolescents are estimated to experience neglect and/or physical, psychological or sexual abuse[3], and approximately 4.6 million children live in poverty[4]. Although a growing literature links child maltreatment (CM) to several poor health outcomes[5–7] in childhood through to older ages, evidence on links to mortality in adulthood is based primarily on adversity scores[8–12], which include other experiences such as living in a household with someone who has previously been imprisoned. While a more extensive literature including systemic reviews[2] demonstrates associations for early-life socioeconomic disadvantage, few studies[9] consider both CM and early-life socioeconomic disadvantage simultaneously in relation to mortality in adulthood. This omission is important because, although these ELAs are related, they represent distinct concepts[13] with potential differences in mortality risk. Clarifying the extent to which CM associates with later mortality independent of childhood socioeconomic background (and vice versa) would inform understanding of the role of different types of early-life exposures.

Moreover, it is possible that associations vary for *specific* types of CM and mortality in adulthood, given the differences reported for several outcomes in adulthood, including socioeconomic disadvantage[14,15], mental[16–18] and physical[6] health. Variation in associations with adult mortality cannot be determined from the sparse literature available to date for specific types of CM. One US study that combined multiple CMs together, found no risk of premature mortality although follow-up was limited to young adulthood[19]. In a second US study, associations were found in women but not men for physical and psychological abuse with all-cause mortality over 20-years of follow-up from ages 25y to 74y at baseline; information on sexual abuse and neglect was unavailable[9]. Thus, previous studies have investigated CM as a combined score of different types[9,19] or a limited number of types examined separately[9]. With such limitations of research conducted to date, possible differential associations for specific types of CM (i.e. their independence from each other as well as from early-life socioeconomic disadvantage) on mortality in adulthood are not well understood.

With respect to potential pathways from ELAs to adult mortality, it is well-established that, for example, CM is associated with detrimental factors in adulthood, including socioeconomic circumstances[14,15], risky health behaviors (e.g. smoking, drug misuse, problem drinking)[17,20,21], obesity[6], poor mental [16–18] and physical health[22]. In turn, these factors are linked to mortality[23–25]. Yet existing literature examining such intermediaries is limited, particularly in relation to CM. Understanding pathways

For peer review only - http://bmjopen.bmj.com/site/about/guidelines.xhtml

through which specific types of ELAs link to mortality in mid-adulthood is important for developing appropriate interventions that aim to reduce inequalities in mortality.

Given current knowledge gaps, we aimed to establish in a general population sample followed from birth: (i) the extent to which CM and early-life socioeconomic disadvantage are associated independently with premature mortality in midlife (from 44/45y to 58y) and whether associations vary by type of ELA; and (ii) whether associations are explained by potential intermediaries including adult socioeconomic, behavioural, adiposity, mental health and cardio-metabolic status.

Methods

The 1958 British birth cohort consists of over 17,000 participants followed-up since birth during one week of March 1958[26]. Respondents in mid-adulthood are broadly representative of the surviving cohort[27]. Ethical approval was given, including at 50y by the London Multicenter Research Ethics Committee and participants gave informed consent at various sweeps. Of 11,971 invited at 44/45y, 9,310 completed at least one question on CM and had information on mortality (44/45-58y) (see supplementary figure 1).

Early-life adversities: Socioeconomic disadvantage was identified from prospectively recorded information on father's occupation at the participant's birth. Those with a father in an unskilled manual occupation were classified as disadvantaged. Child neglect was identified from prospectively collected information at 7y and 11y from interview questions to the child's mother and teacher. Eleven indicators of neglect were selected to represent conventional definitions and were summed to create a score (range 0-11). A score ≥3 yielded a prevalence estimate in line with other UK estimates[1,3], and was used here to define child neglect. Childhood (0-16y) physical, psychological, witnessing and sexual abuse was reported retrospectively at 44/45y using a confidential computer-assisted data-entry questionnaire. Child neglect and abuse measures have been used in several previous studies that, reassuringly, provide extensive evidence of construct validity[28]. Details of all ELAs are given in Table 1.

All-cause mortality: Information on deaths between 2002/3 and end of 2016 was ascertained from a variety of sources, mostly (N=296) through receipt of death certificates (including date of death) from the National Health Service Central Register. Information from relatives or close friends during survey activities/cohort maintenance allowed identification of 16 further deaths (details in Table 2 footnotes). Cause-specific data were not available.

For peer review only - http://bmjopen.bmj.com/site/about/guidelines.xhtml

Covariates: were selected a-priori. All were prospectively recorded, including maternal age at birth, birthweight (adjusted for gestational age), birth order and 7y physical or cognitive impairment. Additional covariates for CM analyses included social class at birth and household factors (amenities, tenure and crowding) at 7y (details in Figure 1 footnotes). Birthweight was ascertained from clinical records; parents reported all other factors.

Potential mid-adult intermediary factors: were selected based on established associations with both ELAs and premature mortality. Details of included factors are given in Table S1 (supplementary file), i.e. for adult (i) socioeconomic factors: 33y social class and educational qualifications, (ii) behavioural factors: 42y smoking, 45y problem drinking and 42y illegal drugs use in the last 12 months; (iii) adiposity: 45y obesity and waist-hip ratio; (iv) 42y mental health; and (v) cardio-metabolic factors: 45y glycated haemoglobin (HbA1c), triglycerides and low-density lipoprotein cholesterol (LDL-c), all adjusted for medications. Cardio-metabolic factors, height, weight, hip and waist were measured by trained professionals; other factors were self-reported. Most considered intermediaries were associated with mortality in this cohort (Table S2).

Statistical analysis

Cox proportional hazard models were used to estimate hazard ratios and 95% confidence intervals (HR(95%CI)) for associations between each type of ELA and mortality. Survival time included the time from completion of the 44/45y questionnaire to the date of death, censoring (last date of contact) or the end of the study period (December 2016), whichever came first. Schoenfeld residuals were examined to test the assumption of proportional hazards for covariates and potential intermediaries; none violated the assumption (p-values≥0.12).

We examined associations between each type of ELA and mortality in separate analyses for men and women and also tested whether associations differed using an interaction term (i.e. type of ELA and mortality by sex) in analyses of both sexes combined. There was little evidence of effect modification ($p_{sex*ELA}$≥0.28 and Table S3), hence in a first level of analyses we adjusted for sex (model 1). Second, to assess whether associations were independent of other early-life factors, we additionally adjusted for covariates listed above (model 2). Third, because different types of ELAs often cluster[29], we assessed two-way correlations between examined ELAs (Table S4). Most ELAs were weakly or only modestly

For peer review only - http://bmjopen.bmj.com/site/about/guidelines.xhtml

correlated (phi coefficient≤0.50). Therefore, in model 3, we adjusted for all types of ELA simultaneously. For associations that remained in model 3, we assessed the role of potential intermediaries (socioeconomic, behavioural, adiposity, mental health and cardio-metabolic factors) in explaining ELA—mortality associations, by further adjusting model 3 for each potential intermediary (in groups as well as for each factor separately).

In sensitivity analyses, we checked whether restricting the sample to those completing the CM questions at 44/45y affected results, by repeating analyses using the larger sample available for child neglect and socioeconomic disadvantage (N=15,092). Survival time included the time from completion of the 11y survey to the date of death, censoring or the end of the study period, whichever came first. As an additional check on the independence of associations for different types of ELAs from model 3, we examined associations with mortality for groups with only one specific type of ELA vs no ELA.

Missing data ranged from 0.02% (physical, sexual and witnessing abuse) to 21% (LDL-c) (Table S5). Data loss was minimized, by imputing missing data on all substantive model variables (i.e. all variables included in models 1, 2 and/or 3) using multiple imputation chained equations. Imputation models included all substantive model variables and main predictors of missingness[27]. Regression analyses were run across 20 imputed datasets and overall estimates obtained. Imputed results were similar to those obtained using observed values (Table S6); the former are presented.

Patient and public Involvement

Patients and the public were not involved in the design of the study, or in the interpretation or writing up of the manuscript.

Results

The prevalence of ELAs varied from 1.6% (sexual abuse) to 11% (psychological abuse) with 10% classified as socioeconomically disadvantaged in early-life (Table 2). The majority of participants reported no ELA (71%) with 19% reporting one and 10% reporting two or more types of ELA. Between 44/45y and 58y, 3.4% of the sample died (N=312).

All types of ELA were associated with risk of death (44/45y-58y) after controlling for covariates (Model 2; Figure 1 and Table S6), for example HR for neglect was 1.49(1.08,2.07) and for physical abuse was 2.15(1.54,3.02). In models simultaneously adjusted for all other types of ELA (Model 3) associations remained for all except psychological and witnessing abuse, namely for neglect (HR:1.43(1.03,1.98)),

For peer review only - http://bmjopen.bmj.com/site/about/guidelines.xhtml

physical abuse (HR:1.73(1.11,2.71)), sexual abuse (HR:2.64(1.52,4.59)) and socioeconomic disadvantage (HR:1.93(1.45,2.58)). The reduction in HRs between Models 2 and 3 was seen consistently for all ELAs, although modest in some instances, for example, for early-life socioeconomic disadvantage the HR reduced from 2.12(1.60,2.82) to 1.93(1.45,2.58) after adjusting for all CMs.

In regard to potential intermediaries, associations between ELAs and death in mid-adulthood were largely unaffected by adjustment for the range of factors examined (Table 3). However, most associations attenuated after adjustment for adult health behaviors, for example HRs for physical abuse attenuated from 1.73(1.11,2.71) to 1.50(0.96,2.34). Separate adjustment for each health behavior in turn showed a predominant attenuating effect of smoking (Table S7). Additionally, associations for neglect and early-life socioeconomic disadvantage attenuated after controlling for adult socioeconomic factors. For sexual abuse and early-life socioeconomic disadvantage reductions in the strong associations with mortality in mid-adulthood were negligible after accounting for intermediaries.

In sensitivity analysis using the larger sample available for child neglect and socioeconomic disadvantage (N=15,092), main findings were largely unaltered to those presented in Figure 1 (Table S8). Checks on the independence of associations performed for groups with only one specific type of ELA (vs no ELA) showed broadly similar mortality associations (albeit with wider confidence intervals) to main results in Figure 1 model 3 (Table S9).

Discussion

In this large population-based study on different types of ELA and mortality in mid-adulthood we showed several important findings. First, some ELAs, but not all, were associated with higher risk of premature mortality in mid-adulthood. That is, findings varied by type of adversity. Child sexual abuse was strongly associated with mortality with a 2.6 times higher risk of premature death in mid-adulthood, although it was the least prevalent adversity. For early-life socioeconomic disadvantage, experienced by 10% of the population, there was an approximate doubling in risk of premature mortality. For physical abuse and neglect the estimated elevated risk of death was more modest (73% and 43% higher respectively), whereas no associations were observed for psychological and witnessing abuse. Second, observed associations were independent of potential confounding factors and the other adversities examined. Importantly, the specific CM associations were mostly robust when accounting for early-life socioeconomic disadvantage and vice versa. Third in relation to potential intermediaries, associations for all types of ELA attenuated after

For peer review only - http://bmjopen.bmj.com/site/about/guidelines.xhtml

controlling for adult health behaviours, in particular smoking. But, in some instances this attenuation was minor, such that for sexual abuse the association was largely unaltered. Associations for early-life socioeconomic disadvantage and neglect were also attenuated by adult socioeconomic factors. Other examined intermediaries including cardio-metabolic markers did little to explain observed associations between specific CMs and mortality in mid-adulthood or for early-life socioeconomic disadvantage.

Our study has several notable strengths. The range of data available on different types of ELA facilitated simultaneous analysis to inform on their independent effects. This is essential for investigating distinct effects of CM on mortality, i.e. separate from those of socioeconomic background and also, in regard to specific types of CMs. Inclusion of child neglect is particularly important given that it is often ignored in research on CM[30]. A follow-up of approximately 14y is a further study strength, as is use of linked mortality data, which is independent of ongoing study participation. Alongside the 14y mortality follow-up there are advantages of using a single-age sample in reducing the range of possible causes of premature death and related underlying pathways. However, study limitations are acknowledged. Ascertainment of childhood maltreatment is not straightforward, with limitations noted for all methods[1], including those used here. While child neglect indicators were measured prospectively and included many aspects of the conventional definition (e.g. failure to ensure a child's basic physical, emotional and educational needs), there were some omissions (e.g. failure to ensure a child's safety) and neglect after age 11y may be missed. However, our measure uses information from different sources (parents and teachers) and at two ages (7y and 11y) which may reduce misclassification and rather than relying on individual items, we used a composite score. Abuse by a parent (up to 16y) was reported retrospectively and does not include abuse by others possibly leading to an underestimate of prevalence. Nonetheless, prevalence estimates of CM were generally in keeping with previous approximates for the UK[1,3]. An exception is child sexual abuse where prevalence is low and estimates may be under-powered. Thus, we have used both prospective (neglect and early-life socioeconomic disadvantage) and retrospective (abuse) measures and we acknowledge that these may identify different groups of individuals[31]. However, it is reassuring that a broad range of studies based on our measures of child neglect and abuse provide extensive evidence of construct validity[28]. As with any long-term study, selection bias needs to be considered: by 45y, when information was collected on child abuse, not all in the cohort had survived (6.7% had died); however over half of these deaths had occurred before 7y (mostly in the first months of life)[27]. Selection bias may affect findings reported here, but only if patterns of association with mortality differ in the surviving and deceased populations. Relatedly, sensitivity analysis for child neglect and socioeconomic disadvantage in a larger sample with longer follow-up from 11y suggests that study results are robust. The analytic approach used allows insights into possible mechanisms underlying ELA associations with premature mortality, but it

For peer review only - http://bmjopen.bmj.com/site/about/guidelines.xhtml

is not possible to determine the most important intermediaries without considering confounding between mediator—outcome associations or the interrelationship between the mediators. Mortality data were available till age 58y and thus results apply to premature mortality; we are unable to infer whether associations will be stable through to later life. Finally, cause-specific mortality data was unavailable, restricting understanding of possible mechanisms linking ELAs to different causes of premature death.

Our main finding of varying associations for specific types of ELAs with risk of premature mortality is novel largely because there is a dearth of literature that focuses on such variations. The large population and range of ELA measures examined in our study compared with two previous studies[9,19] has facilitated this novel finding. Notably, in respect of CMs, we found that after accounting for confounders and all other ELAs, sexual and physical abuse and also neglect were independently associated with elevated mortality in mid-adulthood in the 1958 birth cohort, but there were no associations for psychological and witnessing abuse. The strong association for child sexual abuse, 2.6 times higher risk of premature mortality, is particularly important given the lack of evidence to date. One previous study of CM and mortality did not include sexual abuse[9] and a second study considered sexual abuse, physical abuse and neglect as a composite measure[19]; thus comparison with our findings for specific types of CMs is not possible. The latter composite measure study reported no association with mortality in young adulthood[19] whereas, our focus is on premature mortality for the age range 44/45y to 58y. The life-stage examined might explain discrepant findings i.e. associations with mortality were not present in young adulthood[19] but may emerge by mid-adulthood as suggested here. For physical abuse, our finding of a 73% higher risk of premature mortality is consistent with a previous estimate of 58% higher risk of death for severe physical abuse in US women aged over 45y to 94y at the end of follow-up[9]. This broad similarity in estimates for physical abuse was unexpected given the wider age range of US study participants compared with our range 44/45y to 58y for mortality follow-up. Nonetheless, there was a discrepancy between our observed association for physical abuse and mortality and the lack of an association in US men[9]. The most common cause of death for men between 20y to 49y is due to external causes (e.g. accidents and suicides), whereas, from 50y, cancer, heart disease and strokes and respiratory diseases are the most common causes of death[32]. These variations in main cause of death may explain the noted discrepancy in findings. For child neglect we are unable to compare our finding of an independent association, with a 43% higher risk of premature death in mid-adulthood, as neither of the two previous CM—mortality studies investigated this exposure separately[9,19]. Thus, our finding provides new evidence for an important component of CM where knowledge of long-term outcome is particularly sparse[30]. For witnessing abuse in childhood we are not aware of any previous study with which to confirm our null finding in relation to premature mortality; whereas for psychological abuse, findings for the US (weak association in women

For peer review only - http://bmjopen.bmj.com/site/about/guidelines.xhtml

only[9]) and our UK (null) study are discrepant. Possible reasons for discrepancies include differences in age at death, abuse measurement and also, the extent to which other ELAs were taken into account. In respect of the latter, it is noteworthy that our findings for specific CM associations with elevated mortality in mid-adulthood were independent of other types of CM as well as childhood socioeconomic circumstances, highlighting the potential for long-term harm associated with specific CMs.

A further novelty of our study is the demonstration that the early-life socioeconomic disadvantage association of an approximate doubling in risk of premature all-cause mortality was independent of specific CMs. While links between early-life socioeconomic disadvantage and mortality in adulthood are well-established[2] and consistent with previous work in this cohort[33], few studies[9] consider both CM and early-life socioeconomic disadvantage simultaneously. By suggesting that, notwithstanding the utility of understanding the long-term impact of CM, the latter does not appear to undermine or explain the strong and robust findings relating to childhood socioeconomic disadvantage our study adds new knowledge to the literature. This is important in a policy context as the recent emphasis on adverse childhood experiences may displace attention away from the early socioeconomic environment, as argued elsewhere[13].

Our findings suggest that adult smoking may be a consistent and in some instances important explanatory factor across observed associations. This was expected because smoking remains one of the most common preventable causes of premature death in adults[34]; and, CM[6,21] and early-life socioeconomic disadvantage[35] are associated with subsequent smoking patterns. Thus, interventions to reduce smoking prevalence in specific ELA groups, either by reducing initiation or promoting cessation, might be considered as possible strategies to lessen differences in premature mortality. Interestingly, while specific CMs in this cohort were associated with the wide range of potential intermediary factors examined, these did not appear to explain associations with mortality. In particular, the strong association for sexual abuse was little explained by examined factors. Nonetheless, the potential intermediary factors considered here may play a role in pathways to mortality at older ages. Whereas in relation to the focus here on premature mortality, further insight into pathways from sexual abuse and other ELAs might be gained in future studies of cause-specific mortality.

In summary, our findings of independent associations for specific types of CM (sexual and physical abuse and neglect) and early-life socioeconomic disadvantage with increased risk of premature mortality in mid-adulthood highlight the long-lasting consequences of these ELAs. Smoking may be a particularly important intermediary for physical abuse, neglect and early-life socioeconomic disadvantage associations; adult socioeconomic factors may be an additional intermediary for neglect and early-life socioeconomic

For peer review only - http://bmjopen.bmj.com/site/about/guidelines.xhtml

disadvantage. These findings are relevant for public health because, for example, an estimated 3.1 million adults in England and Wales reported being victims of sexual abuse before 16y[36] and approximately 4.6 million children in the UK live in poverty[4]. Moreover, relative child poverty is projected to rise from 29.7% to 36.6% in the UK between 2018 to 2022[37]. Given these stark projections and our study findings of a strong relationship between childhood disadvantage and an early adult death, policies focused on improving socioeconomic opportunities and assistance to adopt and maintain positive health behaviors for individuals from disadvantaged backgrounds may reduce the burden of premature mortality.

Conclusions

In sum, our findings highlight the potential of specific types of CMs (i.e. sexual abuse, physical abuse and neglect) for long-term harm. Notwithstanding this important finding, childhood socioeconomic disadvantage associations with premature mortality are strong and not explained by associations with CM.

Abbreviations

CM: Child maltreatment

ELA: Early-life adversity

For peer review only

For peer review only - http://bmjopen.bmj.com/site/about/guidelines.xhtml

Contributors

CP and SMPP conceived the study. NTR carried out the analysis and drafted the paper. All authors contributed to the interpretation of data, revision of the manuscript, and approved its final version.

Declaration of interests

We declare no competing interests.

Acknowledgments

Application for all-cause mortality was made through the UK Data service. This work was supported by US National Institute on Aging (NIA) of the National Institutes of Health under award number U24AG047867 and the UK Economic and Social Research Council (ESRC) and the Biotechnology and Biological Sciences Research Council (BBSRC) and by the National Institute for Health Research Biomedical Research Centre at Great Ormond Street Hospital for Children NHS Foundation Trust and University College London. SMPP is funded by a UK Medical Research Council Career Development Award (ref: MR/P020372/1). The views expressed in the publication are those of the authors and not necessarily those of the funders. The funders had no input into study design; data collection, analysis, and interpretation; in the writing of the report; and in the decision to submit the article for publication. Researchers were independent of influence from study funders.

Ethical declarations

Ethical approval was given, including at 50y by the London Multicenter Research Ethics Committee. Participants gave informed consent at various sweeps.

Data sharing statement Cohort data comply with ESRC data sharing policies, readers can access these data via the UK Data Archive at http://www.data-archive.ac.uk/

For peer review only

Table 1. Definition of early-life adversities (child maltreatment and early-life socioeconomic disadvantage) and representative variables from the 1958 British Birth cohort

	Definition[a]	1958 cohort variables	Age of ascertainment (method)[b]
Prospective report, birth to 11y			
Socioeconomic disadvantage (birth)		Based on father's occupation at birth[c], using the Registrar General's Classification. Fathers with an unskilled manual occupation or households with no male head were classified as disadvantaged.	Birth (P)
Neglect[d] (7y & 11y)	Failure to meet a child's basic physical, emotional, medical/dental, or education need; failure to provide adequate nutrition, hygiene, or shelter; or failure to ensure a child's safety	- Child looks undernourished, scruffy or dirty<br>- Mother never/hardly ever takes child out[e]<br>- Father never/hardly ever takes child out[e]<br>- Mother shows little/no interest in child's educational progress<br>- Father shows little/no interest in child's educational progress<br>-Mother and Father never/hardly ever read to, or reads with child	7 & 11y (T)<br>7 & 11y (P)<br>7 & 11y (P)<br>7 & 11y (T)<br>7 & 11y (T)<br>7y (P)
Retrospective report at 44/45y			
Physical abuse (0-16y)	Intentional use of physical force or implements against a child that results in, or has the potential to result in, physical injury.	I was physically abused by a parent – punched, kicked or hit or beaten with an object, or needed medical treatment	45y (S)
Psychological abuse[f] (0-16y)	Intentional behaviour that conveys to a child that s/he is worthless, flawed, unloved, unwanted, endangered, or valued only in meeting another's needs.<br>*UK definition[g] includes harmful (unintentional) parent-child interactions: 'the persistent emotional maltreatment of a child such as to cause severe and persistent adverse effects on the child's emotional development'*	- I was verbally abused by a parent (or parent-figure)<br>- I suffered humiliation, ridicule, bullying or mental cruelty from a parent (or parent-figure)<br>- Mother (or mother-figure) and father (or father-figure) were not at all affectionate	45y (S)
Witnessing abuse (0-16y)	Any incident of threatening behaviour, violence, or abuse (psychological, physical, sexual, financial, or emotional) between intimate partners or adult family members, irrespective of sex or sexuality	I witnessed physical or sexual abuse of others in my family	45y (S)
Sexual abuse (0-16y)	Any completed or attempted sexual act, sexual contact, or non-contact sexual interaction with a child by a caregiver	I was sexually abused by a parent (or parent-figure)	45y (S)

a: Gilbert et al. Lancet. 2009;373; b: (S): self-report; (T): teacher-report; (P): parent-report; c: socioeconomic position was classified as missing for fathers' who were unemployed or sick; d:Questions relating to child neglect at 7y and 11y were answered by the child's teacher and mother (or father if the mother was unavailable). The 11 neglect indicators were summed to create a score (range 0–11); those scoring > 3 were classified as neglected (see text for further details); e: e.g. walks, outings, picnics, visits, shopping; [f]In the 1958 cohort psychological abuse was defined as experiencing at least one of the three listed variables; g: Department for Education. Working together to safeguard children. Her Majesty's Government, 2006.

For peer review only - http://bmjopen.bmj.com/site/about/guidelines.xhtml

Table 2. Prevalence of early-life adversities and mortality[a] in the 1958 British birth cohort.

Early-life adversity	Population sample N[b]	Total cases N (%)	Males (%)	Females (%)	Deaths N (%)
Socioeconomic disadvantage	9033	925 (10.2)	9.6	10.8	61 (6.6)
Neglect[c]	8460	878 (10.4)	11.1	9.7	49 (5.6)
Physical abuse	9308	562 (6.0)	5.9	6.1	40 (7.1)
Psychological abuse	9310	1000 (10.7)	8.9	12.6	50 (5.0)
Witnessing abuse	9308	559 (6.0)	4.4	7.6	33 (5.9)
Sexual abuse	9308	149 (1.6)	0.5	2.7	17 (11.4)
Deaths 44/45y-58y[a]	9310	312 (3.4)	3.7	3.0	

[a]Date of death was ascertained through receipt of death certificates to the Centre for Longitudinal Studies from the National Health Service Central Register (N=296) i.e. data missing for 16 individuals (see: National Child Development Study Deaths Dataset, 1958-2016 UK Data Service for details). Using survey/cohort maintenance data we determined if the deceased died between 45-50y (N=7), 50-55y (N=5) and 55-58y (N=4). Date of death was estimated as the mid-point between these ages; [b]N varies due to missing data; [c]Those with complete data on 6 or more of 11 neglect items (as detailed in Power C et al. Longit Life Course Stud. 2020).

For peer review only

Table 3. Early-life adversities and risk of all-cause mortality (Hazard ratio (95% CI)) in adults (44/45y to 58y) adjusted for mid-adult (i) socioeconomic (ii) behavioural, (iii) adiposity, (iv) mental health and (v) cardio-metabolic factors[a] (N=9310).

	Socioeconomic disadvantage	Neglect	Physical abuse	Sexual abuse
Model 3	1.93(1.45,2.58)	1.43 (1.03,1.98)	1.73(1.11,2.71)	2.64(1.52,4.59)
+ mid-adult socioeconomic factors	1.82(1.36,2.43)	1.28(0.91,1.79)	1.74(1.11,2.73)	2.54(1.46,4.42)
+ mid-adult behavioural factors	1.75(1.31,2.34)	1.32(0.95,1.83)	1.50(0.96,2.34)	2.43(1.40,4.23)
+ mid-adult adiposity	1.90(1.42,2.53)	1.39(1.00,1.93)	1.75(1.12,2.73)	2.71(1.56,4.73)
+ mid-adult mental health	1.91(1.43,2.55)	1.37(0.99,1.91)	1.70(1.09,2.64)	2.54(1.46,4.42)
+ mid-adult cardio-metabolic factors	1.89(1.42,2.53)	1.39(1.00,1.93)	1.65(1.05,2.57)	2.71(1.56,4.72)

Model 3 (adjustments shown in Figure 1 footnotes); [a] Models were adjusted for each intermediary group of factors separately (not simultaneously). See details of intermediary factors in Table S1.

For peer review only

References

1. Gilbert R, Widom CS, Browne K, Fergusson D, Webb E, Janson S. Burden and consequences of child maltreatment in high-income countries. Lancet. 2009;373(9657):68–81.

2. Galobardes B, Lynch JW, Davey Smith G. Childhood Socioeconomic Circumstances and Cause-specific Mortality in Adulthood: Systematic Review and Interpretation. Epidemiol Rev. 2004;26(1):7–21.

3. Radford L, Corral S, Bradley C, Fisher H, Bassett C, Howat N, et al. Child abuse and neglect in the UK today. NSPCC, London. 2011.

4. Measuring Poverty 2019: A report of the Social Metrics Commission. The Legatum Institute. 2019.

5. Norman RE, Byambaa M, De R, Butchart A, Scott J, Vos T. The Long-Term Health Consequences of Child Physical Abuse, Emotional Abuse, and Neglect: A Systematic Review and Meta-Analysis. PLoS Med. 2012;9(11).

6. Power C, Pinto Pereira SM, Li L. Childhood Maltreatment and BMI Trajectories to Mid-Adult Life: Follow-Up to Age 50y in a British Birth Cohort. PLoS One. 2015;10(3).

7. Lanier P, Jonson-Reid M, Stahlschmidt MJ, Drake B, Constantino J. Child maltreatment and pediatric health outcomes: a longitudinal study of low-income children. J Pediatr Psychol. 2010;35(5):511–22.

8. Brown DW, Anda RF, Tiemeier H, Felitti VJ, Edwards VJ, Croft JB, et al. Adverse Childhood Experiences and the Risk of Premature Mortality. Am J Prev Med. 2009;37(5):389–96.

9. Chen E, Turiano NA, Mroczek DK, Miller GE. Association of Reports of Childhood Abuse and All-Cause Mortality Rates in Women. JAMA Psychiatry. 2016;73(9):920–7.

10. Bellis MA, Hughes K, Leckenby N, Hardcastle KA, Perkins C, Lowey H. Measuring mortality and the burden of adult disease associated with adverse childhood experiences in England: a national survey. J Public Health (Bangkok). 2015;37(3):445–54.

11. Felitti VJ, Anda RF, Nordenberg D, Williamson DF, Spitz AM, Edwards V, et al. Relationship of childhood abuse and household dysfunction to many of the leading causes of death in adults. The Adverse Childhood Experiences (ACE) Study. Am J Prev Med. 1998;14(4):245–58.

12. Kelly-Irving M, Lepage B, Dedieu D, Bartley M, Blane D, Grosclaude P, et al. Adverse childhood experiences and premature all-cause mortality. Eur J Epidemiol. 2013 Sep;28(9):721–34.

13. Taylor-Robinson DC, Straatmann VS, Whitehead M. Adverse childhood experiences or adverse childhood socioeconomic conditions? Lancet Public Heal. 2018;3(6).

14. Pinto Pereira SM, Li L, Power C. Child Maltreatment and Adult Living Standards at 50 Years. Pediatrics. 2017;139(1).

For peer review only

15. Currie J, Spatz Widom C. Long-Term Consequences of Child Abuse and Neglect on Adult Economic Well-Being. Child Maltreat. 2010;15(2):111–20.

16. Kisely S, Abajobir AA, Mills R, Strathearn L, Clavarino A, Najman JM. Child maltreatment and mental health problems in adulthood: birth cohort study. Br J Psychiatry. 2018;213(6):698–703.

17. Rehan W, Antfolk J, Johansson A, Jern P, Santtila P. Experiences of severe childhood maltreatment, depression, anxiety and alcohol abuse among adults in Finland. PLoS One. 2017;12(5).

18. Geoffroy M-C, Pereira SP, Li L, Power C. Child Neglect and Maltreatment and Childhood-to-Adulthood Cognition and Mental Health in a Prospective Birth Cohort. J Am Acad Child Adolesc Psychiatry. 2016;55(1):33–40.

19. White HR, Widom CS. Does childhood victimization increase the risk of early death? A 25-year prospective study. Child Abuse Negl. 2003;27(7):841–53.

20. Spatz Widom C, Marmorstein NR, Raskin White H. Childhood victimization and illicit drug use in middle adulthood. Psychol Addict Behav. 2006;20(4):394–403.

21. Kisely S, Abajobir AA, Mills R, Strathearn L, Clavarino A, Gartner C, et al. Child Maltreatment and Persistent Smoking From Adolescence Into Adulthood: A Birth Cohort Study. Nicotine Tob Res. 2019;22(1):66–73.

22. Soares AG, Howe LD, Heron J, Hammerton G, Rich-Edwards J, Magnus MC, et al. How does childhood maltreatment influence cardiovascular disease? A sequential causal mediation analysis. Int J Epidemiol. 2021;

23. Walker ER, McGee RE, Druss BG. Mortality in Mental Disorders and Global Disease Burden Implications. JAMA Psychiatry. 2015;72(4):334.

24. Peacock A, Leung J, Larney S, Colledge S, Hickman M, Rehm J, et al. Global statistics on alcohol, tobacco and illicit drug use: 2017 status report. Addiction. 2018;113(10):1905–26.

25. Berrington de Gonzalez A, Hartge P, Cerhan JR, Flint AJ, Hannan L, MacInnis RJ, et al. Body-mass index and mortality among 1.46 million white adults. N Engl J Med. 2010;363(23):2211–9.

26. Power C, Elliott J. Cohort profile: 1958 British birth cohort (National Child Development Study). Int J Epidemiol. 2006;35(1):34–41.

27. Atherton K, Fuller E, Shepherd P, Strachan DP, Power C. Loss and representativeness in a biomedical survey at age 45 years: 1958 British birth cohort. J Epidemiol Community Health. 2008;62(3):216–23.

28. Power C, Li L, Pinto Pereira SM. Child maltreatment (neglect and abuse) in the 1958 birth cohort: an overview of associations with developmental trajectories and long-term outcomes. Longit Life Course Stud. 2020;

29. Dong M, Anda RF, Felitti VJ, Dube SR, Williamson DF, Thompson TJ, et al. The interrelatedness of multiple forms of childhood abuse, neglect, and household

For peer review only

dysfunction. Child Abuse Negl. 2004;28(7):771–84.

30. Stoltenborgh M, Bakermans-Kranenburg MJ, van IJzendoorn MH. The neglect of child neglect: a meta-analytic review of the prevalence of neglect. Soc Psychiatry Psychiatr Epidemiol. 2013;48(3):345–55.

31. Baldwin JR, Reuben A, Newbury JB, Danese A. Agreement Between Prospective and Retrospective Measures of Childhood Maltreatment. JAMA Psychiatry. American Medical Association; 2019 Jun 1;76(6):584.

32. Mortality team. Does our sex affect what we die from? Off Natl Stat. 2016;

33. Power C, Hyppönen E, Smith GD. Socioeconomic position in childhood and early adult life and risk of mortality: a prospective study of the mothers of the 1958 British birth cohort. Am J Public Health. 2005;95(8):1396–402.

34. Doll R, Peto R, Boreham J, Sutherland I. Mortality in relation to smoking: 50 years' observations on male British doctors. BMJ. 2004;328(7455):1519.

35. Tian J, Gall S, Patterson K, Otahal P, Blizzard L, Patton G, et al. Socioeconomic position over the life course from childhood and smoking status in mid-adulthood: results from a 25-year follow-up study. BMC Public Health. BioMed Central; 2019;19(1):169.

36. Child abuse in England and Wales: January 2020. Statistical Bulletin. Office for National Statistics. 2020.

37. Hood A, Waters T. Living standards, poverty and inequality in the UK: 2017–18 to 2021–22 [Internet]. 2017 [cited 2021 Jan 1]. p. Institute for Fiscal Studies. Available from: https://www.ifs.org.uk/publications/10028

Figure Legend:

Figure 1: Model 1: Adjusted for sex only; Model 2: additionally adjusted for maternal age at birth, birthweight (adjusted for gestational age), birth order and 7y physical or cognitive impairment (yes/no). For associations with neglect, physical, psychological, witnessing and sexual abuse (but not for early-life disadvantage) models additionally adjusted for socioeconomic factors: social class at birth (or if missing, at 7y), 7y household amenities (sharing or lack a bathroom, lavatory or hot water), 7y housing tenure (owner/occupier, renter or other), and 7y household crowding (1+ person/room); Model 3: Model 2 plus simultaneous adjustments for all other early-life adversities

For peer review only

For peer review only

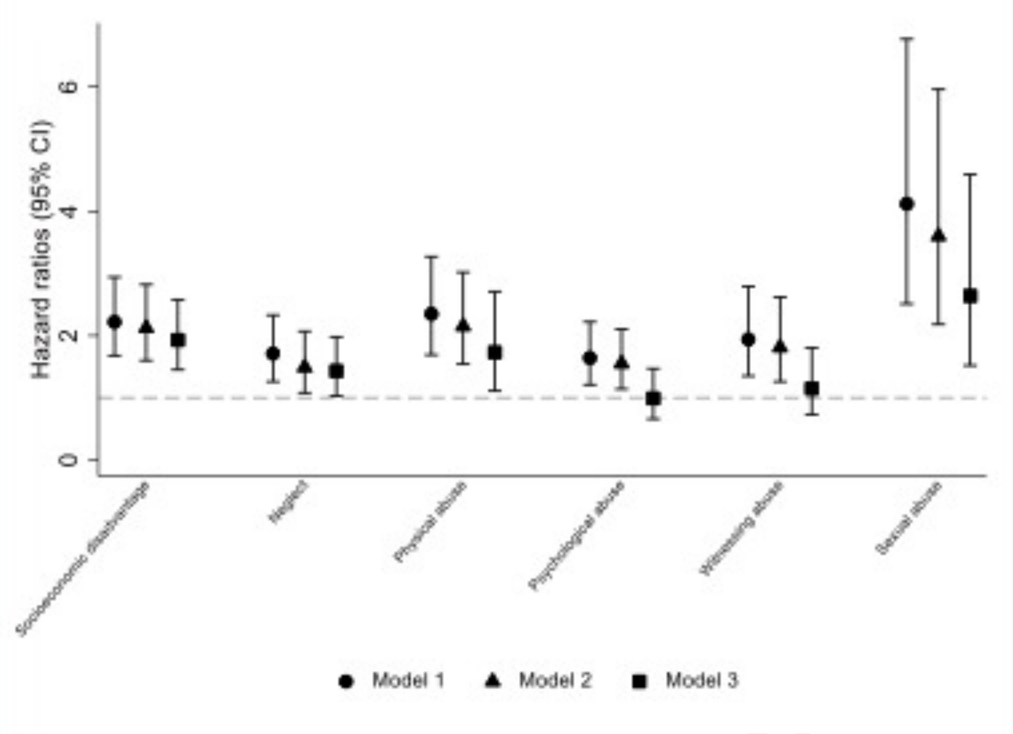
Hazard ratios (95% CI)
0
2
4
6
Socioeconomic disadvantage
Neglect
Physical abuse
Psychological abuse
Witnessing abuse
Sexual abuse
Model 1
Model 2
Model 3

Supplementary tables

Table S1. Distribution of potential intermediary variables (observed values)

Intermediary variables		N(%)/Mean(SD)
**Socioeconomic**		
33y social class[α]		
	I/II	3757 (42.2)
	III non-manual	1938 (21.8)
	III manual	1727 (19.4)
	IV/V	1476 (16.6)
33y educational qualifications		
	None	1765 (21.8)
	O-levels	2797 (34.5)
	A-levels	2424 (29.9)
	Degree	1123 (13.9)
**Behavioural**		
42y smoking		
	Never	4127 (45.8)
	Ex-smoker	2709 (30.1)
	Current	2180 (24.2)
45y problem drinking		
	Low risk	6262 (72.9)
	Risky/hazardous behaviour	1981 (23.1)
	High risk	213 (2.48)
	Almost certainly dependent	131 (1.53)
42y illegal drug use[£]		230 (2.56)
**Adiposity**		
45y obesity		2269 (24.5)
45y waist-hip ratio		0.87 (0.09)
**Mental health**		
42y psychological distress*[€]		2 (0,4)

For peer review only

**Cardio-metabolic factors**[β]	
45y glycated haemoglobin (mmol/L)	5.25 (0.69)
45y triglycerides (mmol/L)*	1.6 (1.1,2.5)
45y low-density lipoprotein cholesterol (mmol/L)	3.45 (0.93)

N varies due to missing data. [α]classes I and II (professional/managerial), class III non-manual (skilled non-manual), class III manual (skilled manual) and classes IV and V (partly/unskilled manual); [£]use of ecstasy, amphetamines, LSD, popper, magic mushrooms, cocaine, temazepan, crack, ketamine, heroin or methadone in last 12 months; *median(inter-quartile range); [€]assessed using the malaise inventory (see Geoffroy et al. PLoS One 2013;8(11) for details); [β]all adjusted for medications (see Li et al. BMJ Open 2019;9(3) for details)

For peer review only

Table S2. Potential intermediary variables and sex-adjusted risk of all-cause mortality (44/45y to 58y; N=9310)

Intermediary variable	HR (95%CI)
**Socioeconomic**	
33y social class [a]	1.21(1.09,1.34)
33y educational qualifications [a]	1.28(1.16,1.41)
**Behavioural**	
42y smoking [a]	3.01(2.32,3.89)
45y problem drinking [a]	3.04(1.65,5.62)
42y illegal drug use	2.59(1.62,4.14)
**Adiposity**	
45y obesity	1.38(1.08, 1.75)
45y waist-hip ratio [b]	2.32(0.44,12.2)
**Mental health**	
42y mental health[c]	1.11(1.07,1.15)
**Cardio-metabolic factors**	
45y glycated haemoglobin (mmol/L)	1.02(1.01,1.03)
45y triglycerides (mmol/L)	1.00(1.00,1.01)
45y low-density lipoprotein cholesterol (mmol/L)	1.00(1.00,1.01)

[a] For categorical variables, extreme category groups are compared (e.g.: lowest vs highest (reference group) social class); [b] per 0.01 unit increase in waist/hip ratio; [c] per increase on 15-point malaise scale

For peer review only

Table S3. Hazard ratios (95% confidence intervals) for early-life adversities in relation to all-cause mortality in 1958 birth cohort participants aged 44/45y to 58y, men and women separately (N=9310)

	Model 1<br>HR (95%CI)	Model 2<br>HR (95%CI)	Model 3<br>HR (95%CI)
		Men	
Socioeconomic disadvantage	1.89 (1.26,2.84)	1.85 (1.22,2.78)	1.69 (1.12,2.57)
Neglect	1.81 (1.22,2.70)	1.49 (0.97,2.30)	1.45 (0.94,2.23)
Physical abuse	2.16 (1.36,3.45)	2.01 (1.25,3.23)	1.81 (1.00,3.29)
Psychological abuse	1.54 (0.98,2.40)	1.46 (0.93,2.28)	0.95 (0.54,1.68)
Witnessing abuse	1.82 (1.04,3.21)	1.66 (0.94,2.95)	1.13 (0.58,2.22)
Sexual abuse	5.41 (2.01,14.58)	5.51 (2.01,15.07)	4.37 (1.53,12.47)
		Women	
Socioeconomic disadvantage	2.60 (1.76,3.85)	2.44 (1.64,3.63)	2.23 (1.49,3.34)
Neglect	1.59 (0.98,2.57)	1.49 (0.89,2.48)	1.41 (0.84,2.35)
Physical abuse	2.57 (1.60,4.12)	2.33 (1.44,3.78)	1.67 (0.85,3.28)
Psychological abuse	1.73 (1.14,2.61)	1.66 (1.09,2.51)	1.02 (0.58,1.79)
Witnessing abuse	2.03 (1.27,3.26)	1.92 (1.19,3.10)	1.17 (0.64,2.14)
Sexual abuse	3.80 (2.15,6.73)	3.21 (1.79,5.75)	2.30 (1.18,4.49)

Adjustments as in Figure 1 footnotes.

For peer review only

Table S4. Correlation between early-life adversities (using Cramér's phi, observed data)

	Socioeconomic disadvantage	Neglect	Physical abuse	Psychological abuse	Witnessing abuse	Sexual abuse
Socioeconomic disadvantage		0.11	0.04	0.05	0.07	0.05
Neglect			0.07	0.04	0.07	0.04
Physical abuse				0.50	0.44	0.21
Psychological abuse					0.39	0.22
Witnessing abuse						0.25
Sexual abuse						

For peer review only

Table S5. Proportion of missing data (ascending order) in sample and distribution of observed and imputed analysis samples

Variable		Missing N(%)	Data distribution	
			Observed sample	Imputed sample*
			%/Mean	%/Mean
Sex		0 (0)		
	Males		49.6	49.6[€]
	Females		50.4	50.4[€]
Psychological abuse		0 (0)	10.7	10.7[€]
Physical abuse		2 (0.02)	6.04	6.04
Sexual abuse		2 (0.02)	1.60	1.60
Witnessing abuse		2 (0.02)	6.01	6.01
45y obesity		61 (0.66)	24.5	24.6
45y waist-to-hip ratio		64 (0.69)	0.87	0.87
Social class[α] at birth		277 (2.98)		
	I/II		19.1	19.1
	III non-manual		10.0	10.0
	III manual		48.7	48.7
	IV/V/No male head		22.2	22.2
42y smoking		294 (3.16)		
	Never		45.8	45.7
	Ex		30.1	30.0
	Current		24.2	24.3
42y Malaise inventory		332 (3.57)	2.46	2.47
42y Illegal drug use		335 (3.60)	2.56	2.60
33y Adult social class[α]		412 (4.43)		
	I/II		42.2	41.7
	III non-manual		21.8	21.7
	III manual		19.4	19.5
	IV/V		16.6	17.1
Maternal age at birth (years)		496 (5.33)	27.5	27.5
45y Problem drinking		723 (7.77)		
	Low risk		72.9	73.2
	Risky/hazardous behaviour		23.1	22.8
	High risk		2.48	2.47

For peer review only

Almost certainly dependent		1.53	1.52
Child neglect	850 (9.13)	10.4	10.5
7y physical or cognitive impairment	1128 (12.1)	4.40	4.72
Birth order	1150 (12.4)		
1st born		38.5	38.8
2nd-4th		54.4	54.0
5th or more		7.17	7.18
7y tenure	1161 (12.5)		
Owner occupied		44.6	44.8
Council rented		37.6	37.5
Private rented		11.9	11.7
Other		5.87	5.99
7y Lacking household amenities	1190 (12.8)	17.0	16.9
33y educational attainment	1201 (12.9)		
None		10.0	10.8
Some		11.8	12.1
O-levels		34.5	34.4
A-levels		29.9	29.3
Degree		13.9	13.5
7y household crowding	1448 (15.6)	39.8	40.0
45y glycated haemoglobin (mmol/L)[£]	1478 (15.9)	165.1	165.2
45y Triglycerides (mmol/L)[£]	1545 (16.6)	52.6	52.9
Birthweight (adjusted for gestational age)[¥]	1581 (17.0)	0.03	0.02
45y low-density lipoprotein cholesterol (mmol/L)[£]	1950 (21.0)	120.1	120.9

*averaged over 20 imputed datasets; [€]No missing data on these variables; [α]classes I and II (professional/managerial), class III non-manual (skilled non-manual), class III manual (skilled manual) and classes IV and V (partly/unskilled manual); [£]glycated haemoglobin and all lipids are presented (and modelled in imputation models) as 100*ln(x) (to ensure data is normally distributed); [¥]standardised scale

For peer review only

Table S6. Hazard ratios (95% confidence intervals) for early-life adversities in relation to all-cause mortality in 1958 birth cohort participants aged 44/45y to 58y

	Model 1 HR (95%CI)	Model 2 HR (95%CI)	Model 3 HR (95%CI)
	Imputed data (N=9310)		
Socioeconomic disadvantage	2.22(1.68,2.94)	2.12(1.60,2.82)	1.93(1.45,2.58)
Neglect	1.71(1.26,2.33)	1.49(1.08,2.07)	1.43(1.03,1.98)
Physical abuse	2.35(1.69, 3.27)	2.15(1.54,3.02)	1.73(1.11,2.71)
Psychological abuse	1.64(1.21,2.22)	1.55(1.14,2.10)	0.99(0.66,1.47)
Witnessing abuse	1.94(1.35,2.79)	1.81(1.26,2.62)	1.15(0.73,1.80)
Sexual abuse	4.12(2.51,6.77)	3.60(2.18,5.96)	2.64(1.52,4.59)
	Complete case analysis[a]		
Socioeconomic disadvantage	2.22(1.68,2.94)	1.99(1.41,2.82)	1.78(1.25,2.54)
Neglect	1.72(1.27,2.34)	1.72(1.16,2.56)	1.68(1.13,2.50)
Physical abuse	2.35(1.69, 3.27)	2.40(1.59,3.64)	1.53(0.88,2.67)
Psychological abuse	1.64(1.21,2.22)	1.81(1.26,2.61)	1.08(0.67,1.75)
Witnessing abuse	1.94(1.35,2.79)	2.51(1.64,3.84)	1.56(0.92,2.62)
Sexual abuse	4.12(2.51,6.77)	5.64(3.10,10.26)	3.70(1.90,7.19)

[a]N varies from 8460 (neglect) to 9310 (psychological abuse) in Model 1 and 6645 (CMs) to 6922 (socioeconomic disadvantage) in Model 3 due to missing data. Adjustments as in Figure 1 footnotes.

For peer review only

Table S7. Hazard ratios (95% confidence intervals) for early-life adversities and risk of premature death (44/45y to 58y) adjusted separately for potential intermediaries[a] (N=9310).

	Socioeconomic disadvantage	Neglect	Physical abuse	Sexual abuse
Model 3[b]	1.96(1.47,2.61)	1.45 (1.04,2.03)	1.72(1.10, 2.70)	2.60(1.49, 4.52)
Socioeconomic				
+ 33y social class	1.89(1.42,2.53)	1.37(0.98,1.91)	1.74(1.11,2.73)	2.57(1.48,4.48)
+ 33y educational qualifications	1.82(1.36, 2.43)	1.29(0.92,1.80)	1.74(1.11,2.72)	2.56(1.48,4.45)
Behavioural				
+ 42y smoking,	1.75(1.31,2.34)	1.29(0.93,1.80)	1.57(1.00,2.45)	2.33(1.34,4.05)
+45y problem drinking	1.91(1.42,2.55)	1.45(1.04,2.01)	1.69(1.08,2.63)	2.78(1.60,4.83)
+42y illegal drug use	1.92(1.44, 2.57)	1.43(1.03,1.98)	1.68(1.07,2.62)	2.61(1.50,4.55)
Adiposity				
+ 45y obesity	1.92(1.44,2.56)	1.42(1.02, 1.97)	1.72(1.10,2.69)	2.63(1.51,4.58)
+ 45y waist-hip ratio	1.89(1.42,2.53)	1.39(1.00,1.92)	1.75(1.12,2.73)	2.73(1.57,4.76)
Mental Health				
+42y psychological distress	1.91(1.43,2.55)	1.37(0.98,1.91)	1.70(1.09,2.64)	2.54(1.46, 4.42)
Cardio-metabolic factors				
+ 45y glycated haemoglobin	1.89(1.41,2.52)	1.39 (1.00,1.93)	1.65(1.05, 2.58)	2.72(1.57, 4.73)
+ 45y triglycerides	1.92(1.44,2.57)	1.42(1.02,1.96)	1.72(1.10, 2.70)	2.67(1.53, 4.64)
+ 45y low-density lipoprotein cholesterol	1.93(1.45,2.58)	1.43 (1.03,1.98)	1.72(1.10, 2.69)	2.61(1.50, 4.54)

[a] For each pathway, models were adjusted for factors separately (not simultaneously). See text and Table S1 for details on intermediary factors.
[b] Model 3 (adjustments shown in Figure 1 footnotes)

For peer review only

Table S8: Hazard ratios (95% confidence intervals) for childhood socioeconomic disadvantage and neglect in relation to all-cause mortality in 1958 birth cohort participants aged 11y to 58y (N=15,092).

	Model 1 HR (95%CI)	Model 2 HR (95%CI)
Socioeconomic disadvantage	1.65(1.40,1.94)	1.55(1.31,1.83)
Neglect	1.72(1.46,2.01)	1.46(1.23,1.73)

Adjustments as in Figure 1 footnotes.

Table S9. Hazard ratios (95% confidence intervals) for distinct non-overlapping early-life adversities (versus none) in relation to all-cause mortality in 1958 birth cohort participants aged 44/45y to 58y.

Early Life adversities vs no Early life adversities	N[a](%)	Model 1 HR (95%CI)	Model 2 HR (95%CI)
No Early life adversities	5968 (78.5)	ref	ref
Socioeconomic disadvantage only	544 (7.16)	2.34(1.63,3.37)	2.30(1.59, 3.32)
Neglect[b] only	522 (7.26)	2.11(1.44,3.08)	2.01(1.34, 3.02)
Physical abuse only	66 (0.87)	1.73(0.60, 5.02)	1.66(0.57, 4.83)
Psychological abuse only	342 (4.50)	1,16(0.63,2.13)	1.17(0.63,2.15)
Witnessing abuse only	108 (1.42)	0.66(0.16,2.66)	0.65(0.16, 2.61)
Sexual abuse only	23 (0.30)	3.62(0.90,14.6)	3.70 (0.91, 15.0)

[a] N varies due to missing data; [b] Those with complete data on 6 or more of 11 neglect items; Adjustments as in Figure 1 footnotes.

For peer review only

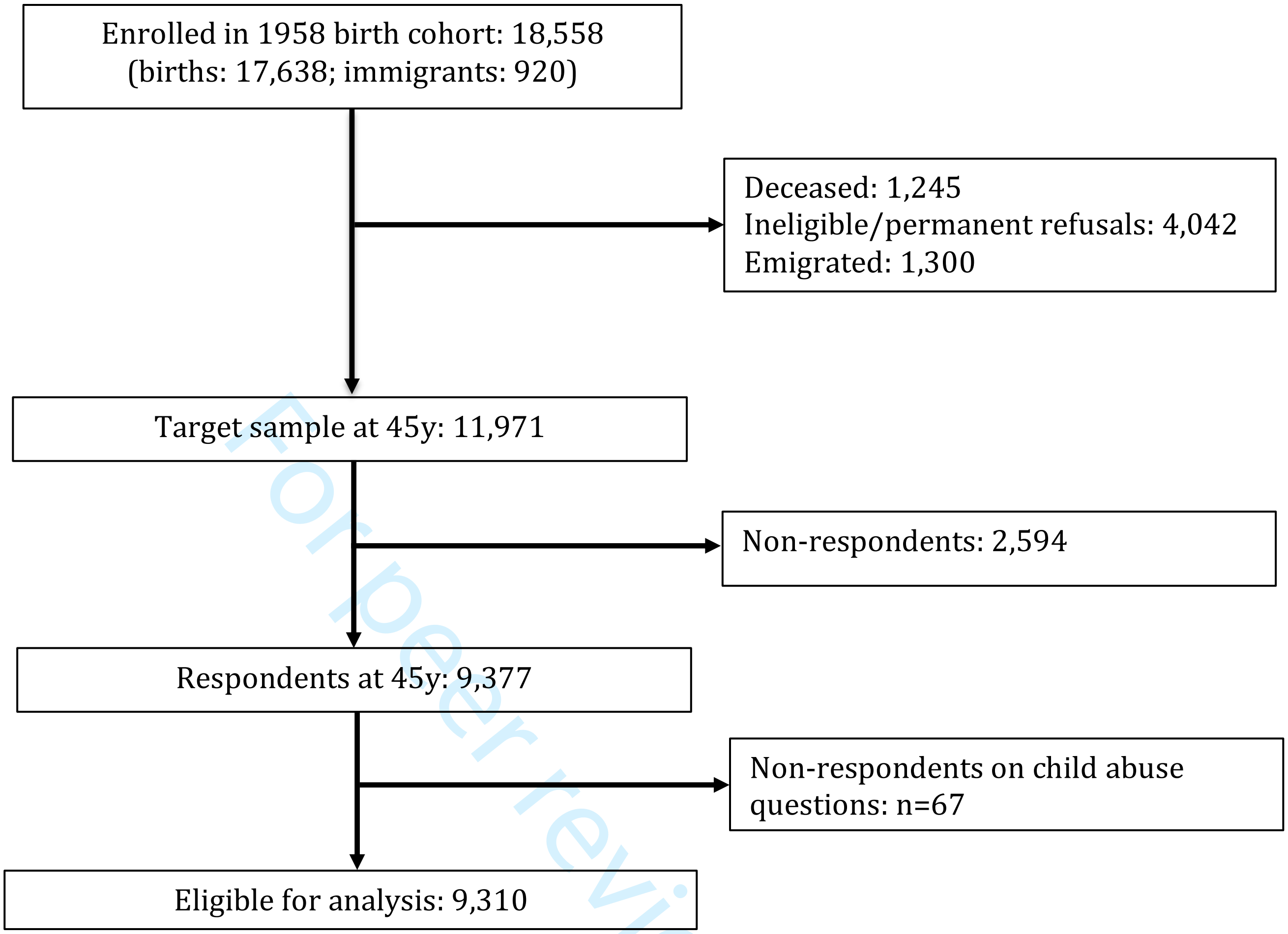

Supplementary Figure 1: Flow diagram of participants from birth who were eligible to be included in analytical sample

For peer review only

STROBE Statement—checklist of items that should be included in reports of observational studies

	Item No	Recommendation	Page No
**Title and abstract**	1	(*a*) Indicate the study's design with a commonly used term in the title or the abstract	1
		(*b*) Provide in the abstract an informative and balanced summary of what was done and what was found	2
**Introduction**			
Background/rationale	2	Explain the scientific background and rationale for the investigation being reported	5
Objectives	3	State specific objectives, including any prespecified hypotheses	6
**Methods**			
Study design	4	Present key elements of study design early in the paper	6
Setting	5	Describe the setting, locations, and relevant dates, including periods of recruitment, exposure, follow-up, and data collection	6
Participants	6	(*a*) *Cohort study*—Give the eligibility criteria, and the sources and methods of selection of participants. Describe methods of follow-up *Case-control study*—Give the eligibility criteria, and the sources and methods of case ascertainment and control selection. Give the rationale for the choice of cases and controls *Cross-sectional study*—Give the eligibility criteria, and the sources and methods of selection of participants	
		(*b*) *Cohort study*—For matched studies, give matching criteria and number of exposed and unexposed *Case-control study*—For matched studies, give matching criteria and the number of controls per case	
Variables	7	Clearly define all outcomes, exposures, predictors, potential confounders, and effect modifiers. Give diagnostic criteria, if applicable	6-7
Data sources/ measurement	8*	For each variable of interest, give sources of data and details of methods of assessment (measurement). Describe comparability of assessment methods if there is more than one group	*6-8*
Bias	9	Describe any efforts to address potential sources of bias	8
Study size	10	Explain how the study size was arrived at	8
Quantitative variables	11	Explain how quantitative variables were handled in the analyses. If applicable, describe which groupings were chosen and why	8
Statistical methods	12	(*a*) Describe all statistical methods, including those used to control for confounding	8
		(*b*) Describe any methods used to examine subgroups and interactions	8
		(*c*) Explain how missing data were addressed	8
		(*d*) *Cohort study*—If applicable, explain how loss to follow-up was addressed *Case-control study*—If applicable, explain how matching of cases and controls was addressed *Cross-sectional study*—If applicable, describe analytical methods taking account of sampling strategy	
		(*e*) Describe any sensitivity analyses	8

Continued on next page

For peer review only - http://bmjopen.bmj.com/site/about/guidelines.xhtml

**Results**			
Participants	13*	(a) Report numbers of individuals at each stage of study—eg numbers potentially eligible, examined for eligibility, confirmed eligible, included in the study, completing follow-up, and analysed	9
		(b) Give reasons for non-participation at each stage	
		(c) Consider use of a flow diagram	
Descriptive data	14*	(a) Give characteristics of study participants (eg demographic, clinical, social) and information on exposures and potential confounders	9
		(b) Indicate number of participants with missing data for each variable of interest	
		(c) *Cohort study*—Summarise follow-up time (eg, average and total amount)	
Outcome data	15*	*Cohort study*—Report numbers of outcome events or summary measures over time	*9*
		Case-control study—Report numbers in each exposure category, or summary measures of exposure	
		Cross-sectional study—Report numbers of outcome events or summary measures	
Main results	16	(*a*) Give unadjusted estimates and, if applicable, confounder-adjusted estimates and their precision (eg, 95% confidence interval). Make clear which confounders were adjusted for and why they were included	9
		(*b*) Report category boundaries when continuous variables were categorized	
		(*c*) If relevant, consider translating estimates of relative risk into absolute risk for a meaningful time period	
Other analyses	17	Report other analyses done—eg analyses of subgroups and interactions, and sensitivity analyses	9
**Discussion**			
Key results	18	Summarise key results with reference to study objectives	9-10
Limitations	19	Discuss limitations of the study, taking into account sources of potential bias or imprecision. Discuss both direction and magnitude of any potential bias	10-11
Interpretation	20	Give a cautious overall interpretation of results considering objectives, limitations, multiplicity of analyses, results from similar studies, and other relevant evidence	11-12
Generalisability	21	Discuss the generalisability (external validity) of the study results	12
**Other information**			
Funding	22	Give the source of funding and the role of the funders for the present study and, if applicable, for the original study on which the present article is based	14

*Give information separately for cases and controls in case-control studies and, if applicable, for exposed and unexposed groups in cohort and cross-sectional studies.

Note: An Explanation and Elaboration article discusses each checklist item and gives methodological background and published examples of transparent reporting. The STROBE checklist is best used in conjunction with this article (freely available on the Web sites of PLoS Medicine at http://www.plosmedicine.org/, Annals of Internal Medicine at http://www.annals.org/, and Epidemiology at http://www.epidem.com/). Information on the STROBE Initiative is available at www.strobe-statement.org.

For peer review only
